# Digital biomarkers derived from wearable and portable devices in prodromal Parkinson’s disease and at-risk populations: a scoping review

**DOI:** 10.3389/fnagi.2026.1850991

**Published:** 2026-10-12

**Authors:** Ting Yu, Qian Wang, Mingxia Pan, Yuanfang Xiong, Shiya Hu, Yiping Deng, Lingyu Kuang, Hanjiao Liu

**Affiliations:** 1 Shenzhen Hospital of Integrated Traditional Chinese and Western Medicine, Shenzhen, Guangdong, China; 2 School of Nursing, Fujian University of Traditional Chinese Medicine, Fuzhou, Fujian, China; 3 Shenzhen Clinical College of Integrated Traditional Chinese and Western Medicine, Affiliated to Guangzhou University of Chinese Medicine, Shenzhen, Guangdong, China

**Keywords:** digital biomarkers, portable devices, prodromal Parkinson’s disease, scoping review, sensors, wearable devices

## Abstract

**Background:**

Prodromal Parkinson’s disease (pPD) represents a pre-diagnostic stage during which subtle and heterogeneous changes may be difficult to detect through conventional clinical assessments. Digital biomarkers derived from wearable or portable devices offer opportunities for objective and longitudinal assessment. This scoping review aimed to map the current evidence on digital biomarkers in individuals with formally defined pPD or populations at increased risk of Parkinson’s disease (PD), focusing on measurement approaches, analytical methods, types of evidence, and methodological gaps.

**Methods:**

A systematic search of PubMed, Web of Science, Embase, IEEE Xplore, Scopus, and ACM Digital Library was conducted from database inception to August 31, 2026. Studies investigating digital biomarkers derived from wearable or portable devices in individuals with formally defined pPD or populations at increased risk of PD were included. Study characteristics, digital biomarker domains, measurement and analytical approaches, and types of evidence were synthesized descriptively.

**Results:**

Forty-three studies were included, primarily involving individuals with iRBD or other high-risk states associated with *α*-synucleinopathies, population-based or broader prodromal-risk cohorts, genetic high-risk populations, and individuals with idiopathic hyposmia/anosmia. Digital biomarkers encompassed motor, speech, sleep/activity, and multidomain features, with motor-related measures, particularly gait and mobility, being the most extensively investigated. Machine-learning approaches were applied across several domains, whereas genuinely multidomain studies remained limited. Most studies provided cross-sectional evidence of group differences or discriminative performance, whereas prospective evidence regarding future PD diagnosis or phenoconversion was less extensive. Considerable heterogeneity was observed in measurement protocols, analytical approaches, and validation strategies.

**Conclusion:**

Wearable- and portable-device-derived digital biomarkers represent promising candidate tools for characterizing and monitoring individuals across the prodromal PD risk spectrum. However, evidence supporting clinical prediction of phenoconversion remains limited by methodological heterogeneity and a lack of sufficient longitudinal and external validation. Future research should prioritize standardized assessment, large, multicenter longitudinal cohorts, independent external validation, and integration with established clinical and biological markers.

## 1 Introduction

Parkinson’s disease (PD) is a progressive neurodegenerative disorder characterized by motor and non-motor symptoms, with pathological features including the degeneration of dopaminergic neurons and axons in the substantia nigra and the formation of Lewy bodies (Li et al., 2026; Kalia and Lang, 2015). With population aging, PD has become one of the fastest-growing neurological disorders worldwide, imposing substantial health and economic burdens that increase with disease progression and severity (Su et al., 2025; Wijers et al., 2024). By the time clinically established PD is diagnosed, substantial neurodegenerative changes may already have occurred, accompanied by marked depletion of striatal dopamine (Lang and Obeso, 2004; Draoui et al., 2020). However, conventional clinical diagnosis relies largely on motor manifestations and has limited ability to identify individuals during the pre-diagnostic phase, thereby increasing interest in characterizing PD during its prodromal stage.

Prodromal Parkinson’s disease (pPD) refers to the pre-diagnostic phase of PD in which individuals have not yet developed clinically established PD but meet established research criteria for prodromal disease, such as the Movement Disorder Society (MDS) research criteria. Importantly, individual risk factors or prodromal features do not necessarily indicate formally defined pPD. Under the MDS research criteria, the probability of prodromal PD is estimated by integrating multiple risk and prodromal markers. Accordingly, individuals with idiopathic REM sleep behavior disorder (iRBD), idiopathic hyposmia/anosmia, or non-manifesting PD-associated genetic mutations should be considered members of PD high-risk or genetic-risk populations unless formal prodromal PD criteria are applied (Berg et al., 2015). Identifying and monitoring individuals across this risk spectrum remains challenging because prodromal manifestations are often subtle and heterogeneous.

Digital health technologies provide opportunities for the objective and continuous measurement of subtle behavioral and physiological changes. Wearable and portable devices, including inertial sensors and smartphones, can capture movement, speech, physical activity, sleep, and other behavioral or physiological signals that can be processed into digital biomarkers (Alonso et al., 2024). Such biomarkers have increasingly been investigated in individuals with formally defined pPD and in populations at increased risk of PD. However, substantial heterogeneity exists across study populations, measurement approaches, analytical methods, and outcomes. Moreover, existing studies provide different types of evidence, ranging from cross-sectional discrimination and clinical or biological associations to longitudinal monitoring and prediction of phenoconversion. These findings cannot be interpreted as equivalent evidence for the identification or prediction of prodromal PD, highlighting the need for systematic mapping of the field.

Therefore, this scoping review aimed to map the evidence on wearable- and portable-device-derived digital biomarkers in individuals with formally defined pPD and populations at increased risk of PD. We characterized the devices and measurement settings, digital biomarker domains and features, analytical and modeling approaches, and evidence regarding cross-sectional discrimination, clinical or biological associations, longitudinal monitoring, and prediction of phenoconversion. We further identified methodological limitations and evidence gaps relevant to the development and validation of digital biomarkers for risk assessment and monitoring during the pre-diagnostic phase of PD. The review protocol was registered on the Open Science Framework on 8 March 2026 (OSF; DOI: 10.17605/OSF. IO/WD6MV).

## 2 Methods

### 2.1 Methodological framework

This scoping review was conducted in accordance with the methodological guidance of the Joanna Briggs Institute (JBI) (Peters et al., 2020) and reported in accordance with the Preferred Reporting Items for Systematic Reviews and Meta-Analyses Extension for Scoping Reviews (PRISMA-ScR) (Tricco et al., 2018) to enhance the transparency and completeness of the review process. All researchers involved in study screening and data extraction received standardized training on the review methodology and screening procedures.

### 2.2 Defining the research questions

Specific research questions were developed in accordance with the objectives of this scoping review:

1. What wearable or portable devices, sensor types, wearing locations, and measurement settings have been used to study formally defined pPD and populations at increased risk of PD?
2. What types of digital biomarkers and specific features have been investigated, and what methods have been used for data processing, feature extraction, statistical analysis, predictive modeling, and machine learning?
3. What evidence has been reported regarding cross-sectional discrimination, clinical or biological associations, classification performance, prediction of future phenoconversion, and longitudinal monitoring using these digital biomarkers, and what are the main methodological limitations and evidence gaps?

### 2.3 Inclusion and exclusion criteria for literature

The inclusion criteria were developed according to the PCC framework for scoping reviews. Eligible studies met the following criteria: (1) Population (P): individuals with formally defined pPD or populations at increased risk of PD, including those with iRBD, idiopathic hyposmia/anosmia (IH), or non-manifesting PD-associated genetic mutations; (2) Concept (C): digital biomarkers derived from wearable or portable devices for the identification, characterization, monitoring, risk stratification, or prediction of pPD or populations at increased risk of PD; (3) Context (C): laboratory, clinical, community, or home-based settings; and (4) Study types: original quantitative studies published as peer-reviewed journal articles, including cohort, cross-sectional, and case–control studies. Only English-language publications were included. The exclusion criteria were as follows: (1) secondary studies; (2) duplicate publications, protocols without original results, or studies without an available full text; (3) conference abstracts, conference papers or proceedings, preprints, and other non-peer-reviewed publications; and (4) studies focused exclusively on treatment response that did not address the review concept.

### 2.4 Search strategy

A systematic literature search was conducted using a combination of controlled vocabulary and free-text terms combined with Boolean operators, across six databases: PubMed, Web of Science, Embase, IEEE Xplore, Scopus, and ACM Digital Library. The search period extended from database inception through 31 August 2026. The search strategy was designed to identify studies investigating digital biomarkers derived from wearable or portable devices in individuals with formally defined pPD or populations at increased risk of PD. The detailed search strategies used for each database are provided in Supplementary Appendix 1.

### 2.5 Literature screening and data extraction

Retrieved records were imported into EndNote 21 for duplicate removal. Two trained researchers independently screened the titles, abstracts, and full texts according to the predefined eligibility criteria. Disagreements were resolved through discussion or consultation with a third researcher. Before formal data extraction, both researchers independently extracted data from 10 randomly selected studies. An agreement rate of ≥90% was required before formal data extraction could proceed. Data were extracted using a standardized form, including study characteristics, population characteristics, digital biomarkers, devices and measurement approaches, analytical methods, evidence types, performance measures, and main findings. Given the heterogeneity among the included studies, the data were synthesized descriptively, and performance metrics were neither pooled nor directly compared.

## 3 Results

### 3.1 Search results

The literature search identified 5,279 records from six electronic databases. After deduplication and title and abstract screening, 51 full-text articles were assessed for eligibility, of which eight were excluded. A total of 43 studies were ultimately included in this scoping review. The study selection process is presented in Figure 1.

**Figure 1:**
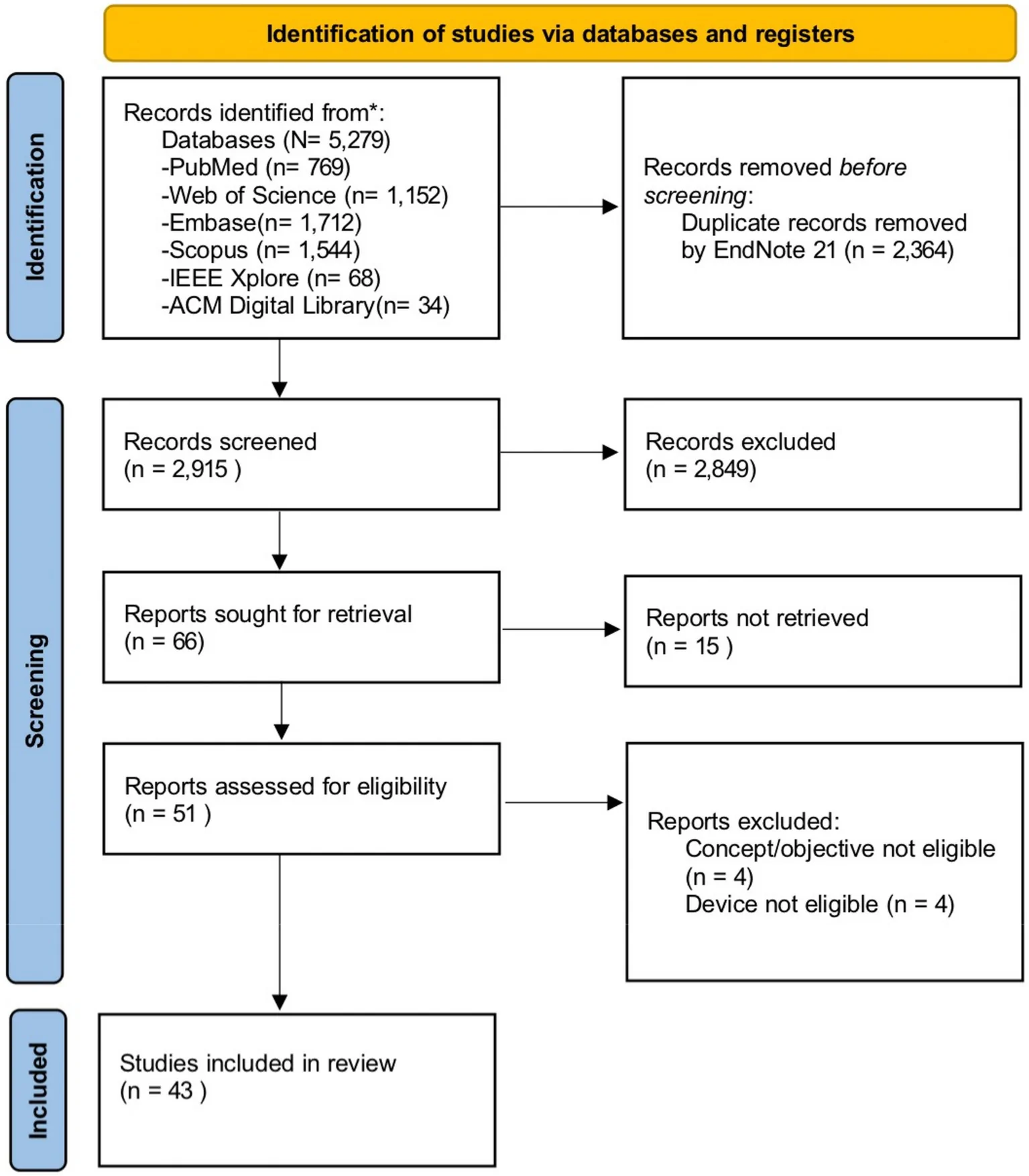
PRISMA-ScR flow diagram of literature search and study selection.

### 3.2 Characteristics of included studies

The characteristics of the included studies are summarized in Table 1. A total of 43 studies published between 2011 and 2026 were included in this scoping review (Guarín et al., 2025; Arora et al., 2018; Arora et al., 2021; Bertram et al., 2026; Bouhadoun et al., 2026; Brink-Kjaer et al., 2023; Rusz et al., 2022; Cavallo et al., 2019; Cen et al., 2024; Cen et al., 2026; Chen et al., 2014; Del Din et al., 2019; Del Din et al., 2020; Elshehabi et al., 2026; Chan et al., 2026; Feng et al., 2020; Hlavnička et al., 2017; Gunter et al., 2025; Gupta and Patel, 2025; Jeancolas et al., 2022; Illner et al., 2024; Skrabal et al., 2022; Ma et al., 2021; Maremmani et al., 2018; Massimi et al., 2026; Mirelman et al., 2016; Mirelman et al., 2011; Ophey et al., 2025; Rovini et al., 2018; Rusz et al., 2018; Pelicioni et al., 2026; Schalkamp et al., 2023; Šimek et al., 2026; Simonet et al., 2021; Stefani et al., 2018; Tzfoni et al., 2026; Winer et al., 2023; Zatti et al., 2024; Winer et al., 2024; Xie et al., 2026; Prasuhn et al., 2021; Sánchez-Rodríguez et al., 2022; Schalkamp et al., 2025). The included studies demonstrated substantial methodological heterogeneity and comprised cross-sectional studies, case–control studies, prospective longitudinal cohorts, and multicenter validation studies (Bertram et al., 2026; Bouhadoun et al., 2026; Del Din et al., 2019; Massimi et al., 2026; Pelicioni et al., 2026; Schalkamp et al., 2023; Schalkamp et al., 2025). The study populations covered a broad spectrum of individuals at increased risk of PD. Based on the primary study population, individuals with iRBD or other α-synucleinopathy-related high-risk states represented the most frequently investigated group, accounting for 27 studies (Guarín et al., 2025; Arora et al., 2018; Arora et al., 2021; Bertram et al., 2026; Bouhadoun et al., 2026; Brink-Kjaer et al., 2023; Rusz et al., 2022; Cen et al., 2024; Cen et al., 2026; Chen et al., 2014; Del Din et al., 2020; Chan et al., 2026; Feng et al., 2020; Hlavnička et al., 2017; Gunter et al., 2025; Jeancolas et al., 2022; Illner et al., 2024; Skrabal et al., 2022; Ma et al., 2021; Massimi et al., 2026; Ophey et al., 2025; Rusz et al., 2018; Šimek et al., 2026; Stefani et al., 2018; Tzfoni et al., 2026; Winer et al., 2023; Zatti et al., 2024). This group was followed by population-based or broader prodromal-risk cohorts (eight studies) (Del Din et al., 2019; Elshehabi et al., 2026; Gupta and Patel, 2025; Pelicioni et al., 2026; Schalkamp et al., 2023; Winer et al., 2024; Xie et al., 2026; Schalkamp et al., 2025), genetic high-risk populations (four studies) (Mirelman et al., 2016; Mirelman et al., 2011; Prasuhn et al., 2021; Sánchez-Rodríguez et al., 2022), and individuals with idiopathic hyposmia/anosmia (four studies) (Cavallo et al., 2019; Maremmani et al., 2018; Rovini et al., 2018; Simonet et al., 2021). Digital biomarkers were primarily acquired using wearable sensors, actigraphy devices, and smartphone-based platforms across laboratory, clinical, and free-living environments.

**Table 1:** Characteristics of the included studies.

Author/year	Country/region	Study design	Population/classification	Sample/comparator	Device/sensor	Setting/task
Guarín et al. (2025)	United States of America (USA)	Cross-sectional	PSG-confirmed iRBD	16 iRBD/32 HCs/18 PD	Standard video camera	Clinical; Finger Tapping Task
Arora et al. (2018)	United Kingdom (UK)	Cross-sectional	PSG-confirmed iRBD	104 iRBD/84 HCs/334 PD	Smartphone; inertial measurement unit (IMU), microphone, touchscreen	Clinic + home (≤7 days); 7 motor tasks
Arora et al. (2021)	United Kingdom	Cross-sectional	PSG-confirmed iRBD	112 iRBD/92 HCs/335 PD	Smartphone microphone	Clinic + home (7 days); sustained vowel/a/
Bertram et al. (2026)	Germany/UK/Denmark	Multicenter observational validation study	PSG-confirmed iRBD	Training: 55 iRBD/23 HCs; Local test: 19 iRBD/12 HCs; external validation: OPDC + PACE	Axivity AX6 wrist-worn triaxial accelerometer	Home/ambulatory; continuous wrist actigraphy, typically 6–7 nights
Bouhadoun et al. (2026)	Canada	Prospective longitudinal	PSG-confirmed iRBD	51 iRBD/89 PD/22 HCs	Smartphone; accelerometer, gyroscope, microphone, touchscreen	Home; daily active motor testing, 12 months
Brink-Kjaer et al. (2023)	United States of America	Cross-sectional	PSG-confirmed iRBD	42 iRBD/21 clinical controls/21 community controls	Wrist actigraph (Axivity AX6)	Home; nocturnal actigraphy ≥7 nights
Rusz et al. (2022)	Czech Republic/Germany	Prospective longitudinal	PSG-confirmed iRBD, stratified by olfactory function	17 iRBD-NOF/30 iRBD-AOF/50 HCs	Head-mounted condenser microphone	Clinic; reading + monologue; 24-month follow-up
Cavallo et al. (2019)	Italy	Cross-sectional	Idiopathic hyposmia (PD high-risk)	30 IH/30 HCs/30 PD	SensHand V1; wearable IMUs	Clinic; 6 upper-limb motor tasks
Cen et al. (2024)	China	Prospective longitudinal cohort	PSG-confirmed iRBD (PD/α-synucleinopathy high-risk)	31 iRBD/33 HCs; 10 converters	MobilityLab wearable inertial sensors	Clinic; normal, fast and dual-task walking
Cen et al. (2026)	China	Prospective longitudinal cohort	PSG-confirmed iRBD (PD/α-synucleinopathy high-risk)	68 iRBD/61 HCs; 21 converters	Opal wearable inertial sensors (APDM)	Clinic; normal, fast and dual-task walking
Chen et al. (2014)	China	Cross-sectional	PSG-confirmed iRBD (PD/α-synucleinopathy high-risk)	24 iRBD/23 HCs	Opal wearable inertial sensor (APDM); tri-axial accelerometer	Clinic; five 30-s standing conditions
Del Din et al. (2019)	Germany	Prospective longitudinal observational	TREND cohort; individuals without PD at baseline, including persons with prodromal/risk markers	696; 16 future PD converters/680 non-converters	DynaPort or Opal wearable inertial sensor; accelerometer	Clinic; 20-m walking under usual-speed, fast-speed and dual-task conditions
Del Din et al. (2020)	United Kingdom	Cross-sectional observational	PSG-confirmed iRBD (PD/α-synucleinopathy high-risk)	63 iRBD/34 HCs	Axivity AX3 wearable tri-axial accelerometer	Free-living; continuous real-world gait monitoring during daily activities
Elshehabi et al. (2026)	Germany	Prospective longitudinal observational	TREND cohort; adults without PD at baseline, including individuals with prodromal/risk markers	1,051 enrolled; 924 survival analysis; 23 future PD/901 non-PD	Opal wearable inertial sensors (APDM); accelerometer and gyroscope	Clinic; 1-min normal-pace walking along 20-m hallway with 180° turns
Chan et al. (2026)	China	Case–control	Video-polysomnography (vPSG)-confirmed iRBD	45 iRBD/45 PD/45 HCs	NeurOptics DP-2000 portable pupillometer; Actiwatch Spectrum Plus	Clinic pupillometry; 1-week free-living wrist actigraphy
Feng et al. (2020)	China	Case–control + prospective nested case–control	vPSG-confirmed iRBD	Study 1: 88 iRBD/44 non-RBD controls/44 converted α-synucleinopathies; Study 2: 22 converters/66 non-converters	Actiwatch Spectrum Plus actigraphy	Free-living habitual daily activity
Hlavnička et al. (2017)	Czech Republic	Cross-sectional	iRBD (PD/α-synucleinopathy high-risk)	50 iRBD/30 *de novo* untreated PD/50 HCs	Head-mounted condenser microphone	Clinic; standardized reading and ~90-s monologue
Gunter et al. (2025)	United Kingdom/Italy	Cross-sectional biomarker classification study	iRBD (PD/α-synucleinopathy high-risk), PD and HCs	93 participants: 49 iRBD/39 PD/5 HCs; 100 DaT scans	Consumer-grade smartphone; accelerometer, microphone & touchscreen	Clinic and home; 7-task ~8-min motor assessment
Gupta and Patel, (2025)	United States	Prospective longitudinal observational	PPMI-defined Prodromal-PD/high-risk, manifest PD and HCs	106 Prodromal-PD/161 Manifest-PD/26 HC; longitudinal subset: 49 Prodromal-PD/27 Manifest-PD/10 HCs	Verily Study Watch; triaxial accelerometer	Free-living home monitoring; continuous habitual wrist movement
Jeancolas et al. (2022)	France	Cross-sectional	iRBD (PD/α-synucleinopathy high-risk), early PD and HCs	41 iRBD/117 early PD/98 HCs	Professional head-mounted microphone; laptop microphone; personal telephone	Clinic + remote telephone recordings; vowels, free speech, syllable repetition, sentence repetition and reading
Illner et al. (2024)	Czech Republic	Cross-sectional	PSG-confirmed iRBD (PD/α-synucleinopathy high-risk), early PD and HCs	21 iRBD/26 early PD/25 HCs	HONOR 9X Lite Android smartphone; built-in microphone	Real-world home monitoring: passive everyday voice calls + active passage reading every 14 days; up to 3 months
Skrabal et al. (2022)	Czech Republic	Cross-sectional	iRBD (PD/α-synucleinopathy high-risk), *de novo* PD and HCs	60 iRBD/60 *de novo* PD/60 HCs; all male	Beyerdynamic Opus 55 head-mounted condenser microphone	Clinic; standardized 80-word passage reading
Ma et al. (2021)	China	Cross-sectional	PSG-confirmed iRBD (PD/α-synucleinopathy high-risk) and HCs	31 iRBD/20 HCs	APDM Mobility Lab; 6 wearable gyroscope and accelerometer sensors	Clinic/laboratory; 10-m corridor walking under usual, fast and dual-task conditions
Maremmani et al. (2018)	Italy	Prospective longitudinal pilot study	Idiopathic hyposmia (PD high-risk population)	20 idiopathic hyposmia; age−/sex-matched HCs used for test–retest/comparison	SensHand v1 and SensFoot v2 wearable inertial sensor systems; accelerometer, gyroscope, magnetometer	Controlled motor tasks: finger tapping, hand opening-closing, pronation-supination, and toe tapping; baseline + 1-year follow-up
Massimi et al. (2026)	United States; multicenter	Retrospective multicenter case–control study	vPSG-confirmed iRBD (PD/α-synucleinopathy high-risk population)	Overall: 99 iRBD/297 controls; actigraphy: 78 iRBD/158 controls	Axivity AX3 or AX6; tri-axial accelerometer	Home-based passive nocturnal monitoring
Mirelman et al. (2016)	Multinational: Israel, United States, Germany, Norway, Spain	Multicenter cross-sectional observational study	Non-manifesting LRRK2-G2019S carriers (genetic PD high-risk), non-carriers, and PD	186 non-manifesting participants: 122 carriers/64 non-carriers; 194 PD: 67 carriers/127 non-carriers	Opal APDM synchronized body-fixed inertial sensors	Lower back + bilateral wrists; 1-min corridor walking under usual and dual-task conditions
Mirelman et al. (2011)	Israel	Cross-sectional observational study	Healthy asymptomatic first-degree relatives of LRRK2-G2019S PD patients; carriers classified as genetic PD high-risk	52 total: 25 LRRK2-G2019S carriers/27 non-carriers	DynaPort lightweight accelerometer	Lower back; 1-min corridor walking under usual, fast and dual-task conditions
Ophey et al. (2025)	Germany	Cross-sectional observational study	Video-PSG-confirmed iRBD; PD/α-synucleinopathy high-risk	68 iRBD/24 HCs	Axivity AX6; 3D accelerometer	Free-living, continuous passive monitoring
Rovini et al. (2018)	Italy	Cross-sectional observational	Idiopathic hyposmia (IH; PD high-risk), PD, and HCs	30 IH/30 PD/30 HCs	SensFoot V2 wearable IMU; accelerometer, gyroscope and magnetometer; 100 Hz	Clinical standardized motor assessment; sensor fixed on dorsum of foot; toe tapping, heel tapping/leg agility, 15-m gait, and 360° rotation
Rusz et al. (2018)	Czech Republic	Cross-sectional observational	vPSG-confirmed iRBD (PD/α-synucleinopathy high-risk), *de novo* untreated PD, and HCs; RBD additionally stratified by MDS prodromal PD criteria	50 RBD/30 *de novo* PD/30 HCs; RBD subgroup: 33 RBD-pPD/17 RBD-A	Sony Xperia Z1 Compact smartphone microphone; 48 kHz, 16-bit	Quiet-room standardized speech assessment; smartphone handheld near ear; sustained /a/, /pa-ta-ka/ repetition, and ~90-s monologue
Pelicioni et al. (2026)	United Kingdom	Prospective population-based cohort/longitudinal follow-up	General population with incident future PD identified during follow-up; prevalent PD also included as reference group	73,413 total: 314 incident PD/119 prevalent PD/72,980 without PD	Wrist-worn accelerometer; UK Biobank accelerometry dataset	Free-living passive monitoring; wrist-worn for 7 consecutive days; followed through linked electronic health records for up to 10 years
Schalkamp et al. (2023)	United Kingdom	Prospective population-based cohort study; machine learning prediction study	General population from UK Biobank; individuals who later developed PD were identified retrospectively based on future clinical diagnosis	Accelerometry subset: 103,712 participants; final prediction analyses included prodromal PD *n* = 113, diagnosed PD *n* = 153, general population controls *n* = 33,009	Wrist-worn accelerometer (UK Biobank Axivity AX3 device)	Real-world free-living movement monitoring; participants wore wrist accelerometers for 7 days; passive assessment of daily movement and sleep behaviors
Šimek et al. (2026)	Czech Republic	Prospective longitudinal cohort study	vPSG-confirmed iRBD (PD/α-synucleinopathy high-risk), early-stage PD, and HCs	71 analyzed: 22 iRBD/23 PD/26 HCs	HONOR 9X Lite smartphone; built-in microphone; 44.1 kHz, 16-bit	Passive real-world speech monitoring during routine phone calls for 24 months
Simonet et al. (2021)	United Kingdom/Switzerland/Italy/Spain/Germany (multicenter)	Cross-sectional case–control study	Recently diagnosed PD; idiopathic anosmia (PD high-risk population); HCs	26 PD/30 idiopathic anosmia/30 HCs	Smartphone video camera; computer vision-based hand tracking (CNN landmark detection)	20-s repetitive finger tapping task; dominant and non-dominant hands; MDS-UPDRS III finger tapping protocol
Stefani et al. (2018)	Austria (multicenter international collaboration)	Cross-sectional case–control study	Video-polysomnography (vPSG)-confirmed iRBD; other sleep-related motor disorders; controls	Total *n* = 90: iRBD *n* = 20; RLS *n* = 20; RLS + SA *n* = 10; untreated SA *n* = 20; HCs *n* = 20	Wrist-worn actigraphy (MicroMini-Motionlogger); tri-axial accelerometer + temperature + light sensors	2-week actigraphy monitoring during habitual sleep; visual pattern recognition of actigraphy plots and quantitative activity analysis
Tzfoni et al. (2026)	Israel	Cross-sectional case–control study	PSG-confirmed isolated REM sleep behavior disorder (iRBD)	Total *n* = 73: iRBD *n* = 15; controls *n* = 58	Lower-back wearable inertial measurement unit (Axivity AX6); tri-axial accelerometer + gyroscope	Participants underwent overnight vPSG followed by six nights of home monitoring using a lumbar wearable sensor
Winer et al. (2023)	United States	Cross-sectional case–control study	Isolated REM sleep behavior disorder (iRBD) and healthy controls (HCs)	iRBD: *n* = 38; matched healthy controls: *n* = 119	Wrist-worn tri-axial accelerometer (Axivity AX6 for iRBD; AX3 for controls)	Continuous free-living 24-h activity monitoring; participants wore accelerometers for ≥14 days
Zatti et al. (2024)	Italy/Germany	Prospective cross-sectional study	PSG-confirmed iRBD without subthreshold parkinsonism; drug-naïve PD; age-matched controls	Total *n* = 148: iRBD *n* = 23/PD *n* = 60/HCs *n* = 65	RehaGait® inertial measurement unit (IMU, Hasomed, Germany); accelerometer + gyroscope	Mobile health technology-instrumented Timed Up-and-Go (TUG) test at normal and fast speed
Winer et al. (2024)	United Kingdom (United Kingdom Biobank cohort)	Prospective longitudinal cohort study	Community-dwelling middle-aged and older adults without baseline PD/AD	*n* = 82,829; incident PD cases = 265 during follow-up; AD cases = 187	Wrist-worn triaxial accelerometer (AX3, Axivity, United Kingdom)	Passive free-living monitoring for 1 week; assessment of 24-h activity/rest-activity rhythms
Xie et al. (2026)	United Kingdom	Prospective longitudinal cohort study	UK Biobank community cohort	UK Biobank community cohort; 383 participants diagnosed with PD > 2 years after accelerometer assessment (classified by the original study as a prodromal PD group); 211 diagnosed at or within 2 years; remaining participants censored as controls	Wrist-worn triaxial accelerometer (Axivity AX3)	Free-living monitoring for 7 days
Prasuhn et al. (2021)	Germany/Italy	Cross-sectional case–control study	Healthy heterozygous Parkin mutation carriers (genetic PD high-risk group) and mutation-free controls	27 healthy heterozygous Parkin mutation carriers; 24 mutation-free controls	Mobility Lab wearable sensor system (APDM); six IMU sensors including accelerometer, gyroscope and magnetometer	Sensor-based posturography and gait analysis under increasing task difficulty conditions
Sánchez-Rodríguez et al. (2022)	Spain	Cross-sectional case–control study	Asymptomatic LRRK2 G2019S mutation carriers (genetic high-risk PD population)	27 asymptomatic LRRK2-G2019S carriers; 36 healthy controls; additionally included 21 idiopathic PD and 20 LRRK2-PD patients for comparison	16 inertial sensors (STT-IWS); accelerometer, gyroscope, magnetometer	Laboratory gait assessment: usual walking, fast walking, dual-task walking
Schalkamp et al. (2025)	United Kingdom/United States (PPMI cohort)	Cohort study with machine-learning modeling	PD patients, healthy controls, and individuals at risk of PD (genetic risk or prodromal symptoms)	PD *n* = 143; HCs *n* = 34; at-risk evaluation cohort *n* = 109	Verily Study Watch (accelerometer, gyroscope, ECG, PPG)	Long-term free-living home monitoring (mean 485 days)

### 3.3 Digital biomarker domains and measurement approaches

An overview of the digital biomarker domains, representative features, and analytical approaches identified across the included studies is presented in Figure 2.

**Figure 2:**
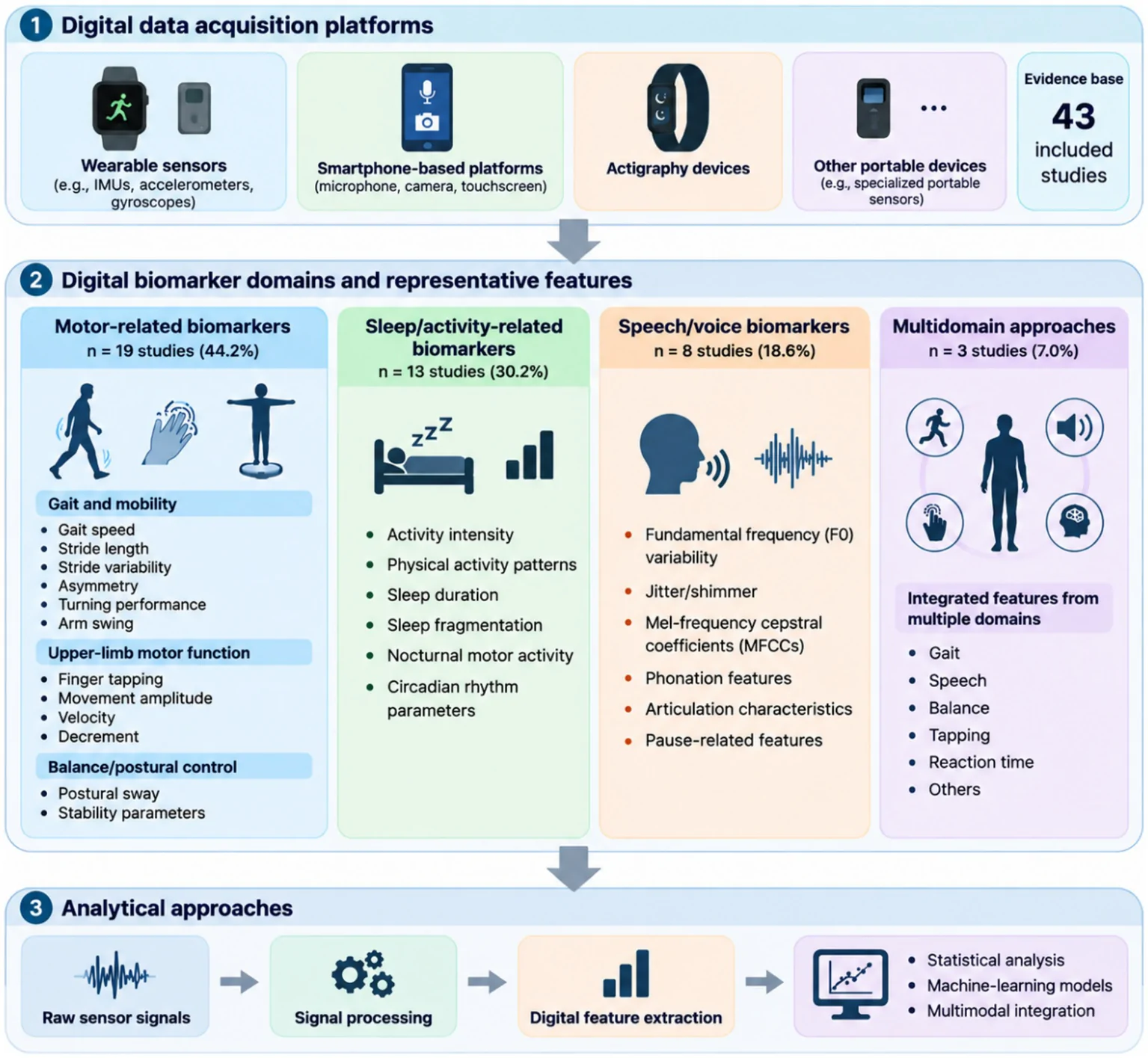
Overview of digital biomarkers across the included studies.

#### 3.3.1 Motor-related digital biomarkers

Motor-related digital biomarkers represented the most extensively investigated domain among the included studies. These measures were primarily obtained using wearable inertial sensors, accelerometers, and smartphone-based platforms and focused on gait and mobility, upper-limb motor function, and balance and postural control. Gait and mobility represented the predominant motor assessment approach. Studies examined spatiotemporal and kinematic features, including gait speed, stride length, gait variability and asymmetry, turning characteristics, arm swing, and trunk movement (Cen et al., 2024; Cen et al., 2026; Del Din et al., 2019; Del Din et al., 2020; Elshehabi et al., 2026; Ma et al., 2021; Mirelman et al., 2016; Mirelman et al., 2011; Rovini et al., 2018; Pelicioni et al., 2026; Zatti et al., 2024; Prasuhn et al., 2021; Sánchez-Rodríguez et al., 2022). Altered gait patterns were reported across individuals with iRBD, genetic-risk populations, and other at-risk populations (Cen et al., 2024; Cen et al., 2026; Del Din et al., 2020; Elshehabi et al., 2026; Ma et al., 2021; Mirelman et al., 2016; Mirelman et al., 2011; Rovini et al., 2018; Zatti et al., 2024; Prasuhn et al., 2021; Sánchez-Rodríguez et al., 2022). Longitudinal evidence further indicated that increased stride-length asymmetry was associated with subsequent PD phenoconversion in individuals with iRBD (adjusted hazard ratio (HR) = 4.47) (Cen et al., 2024). In addition, greater baseline gait variability and asymmetry were associated with a shorter time to PD diagnosis in a population-based cohort (Del Din et al., 2019). Population-based studies also identified gait alterations several years before the clinical diagnosis of PD (Elshehabi et al., 2026; Pelicioni et al., 2026).

Upper-limb motor biomarkers were investigated less frequently and primarily involved finger tapping and repetitive movement assessments. Extracted features included movement amplitude, velocity, amplitude decrement, and temporal characteristics derived from wearable sensors or smartphone-based assessments. Differences in these motor characteristics were reported between individuals at increased risk of PD and control groups (Guarín et al., 2025; Cavallo et al., 2019; Maremmani et al., 2018; Simonet et al., 2021). Balance and postural control biomarkers were investigated in a smaller number of studies and primarily included postural sway, stability, and sensor-derived measures of postural control. Altered postural control characteristics were reported in individuals with iRBD and those with genetic risk compared with control groups (Chen et al., 2014; Prasuhn et al., 2021).

#### 3.3.2 Speech, sleep and activity-related biomarkers

Speech, sleep, and activity-related digital biomarkers represented additional domains investigated in individuals with prodromal features or an increased risk of PD. These biomarkers were primarily acquired using smartphone-based assessments, microphone recordings, and wearable actigraphy devices. Speech-based studies examined acoustic and articulatory features, including fundamental frequency variability, jitter, shimmer, mel-frequency cepstral coefficients, articulation rate, pause characteristics, and voice-quality parameters. Differences in speech characteristics were reported between individuals with iRBD or other high-risk states and control groups. Several studies also applied smartphone-based assessments and machine-learning approaches to discriminate between risk groups (Arora et al., 2021; Rusz et al., 2022; Hlavnička et al., 2017; Jeancolas et al., 2022; Illner et al., 2024; Skrabal et al., 2022; Rusz et al., 2018; Šimek et al., 2026). Sleep-related digital biomarkers were primarily derived from wearable actigraphy and focused on nocturnal movement and sleep-related motor activity. The examined features included movement intensity, activity fragmentation, immobile bouts, twitch activity, sleep efficiency, and nocturnal mobility characteristics (Bertram et al., 2026; Brink-Kjaer et al., 2023; Feng et al., 2020; Massimi et al., 2026; Ophey et al., 2025; Stefani et al., 2018; Tzfoni et al., 2026). Altered nocturnal activity patterns were reported in individuals with iRBD or other high-risk states, and several studies evaluated actigraphy-derived features for discriminating between at-risk individuals and control groups (Bertram et al., 2026; Brink-Kjaer et al., 2023; Massimi et al., 2026; Stefani et al., 2018; Tzfoni et al., 2026). Longitudinal evidence also identified associations between alterations in sleep- and activity-related measures and subsequent PD phenoconversion (Feng et al., 2020). Free-living activity and circadian biomarkers included activity intensity, daily movement patterns, rest–activity rhythms, interdaily stability, intradaily variability, and related measures such as M10 and L5 (Chan et al., 2026; Feng et al., 2020; Ophey et al., 2025; Schalkamp et al., 2023; Winer et al., 2023; Winer et al., 2024; Xie et al., 2026; Schalkamp et al., 2025). Cross-sectional studies identified altered activity or circadian characteristics in at-risk populations (Chan et al., 2026; Ophey et al., 2025; Winer et al., 2023; Schalkamp et al., 2025). In contrast, prospective studies further linked alterations in activity or rest–activity patterns to subsequent PD phenoconversion, risk, or diagnosis, with evidence ranging from longitudinal associations to formal prediction models (Feng et al., 2020; Schalkamp et al., 2023; Winer et al., 2024; Xie et al., 2026).

#### 3.3.3 Multidomain and AI-derived biomarkers

Multidomain digital biomarker approaches, defined in this review as the integration of digital information from two or more distinct functional domains, rather than the use of multiple features or sensors to assess a single domain, incorporated information on motor performance, speech, and other behavioral features. Smartphone-based platforms enabled the simultaneous assessment of gait, voice, dexterity, balance, and other motor or non-motor characteristics. Longitudinal multidomain assessments were also used to characterize changes in individuals at increased risk of PD (Arora et al., 2018; Bouhadoun et al., 2026; Gunter et al., 2025). Machine-learning and AI-based approaches were applied in both single-domain and multidomain studies for digital feature extraction, classification, or prediction. A range of supervised learning methods, including random forests, support vector machines, gradient-boosting algorithms, and neural networks, were used to characterize or discriminate between at-risk populations and, in some studies, to predict future PD-related outcomes (Guarín et al., 2025; Arora et al., 2018; Arora et al., 2021; Bertram et al., 2026; Brink-Kjaer et al., 2023; Cavallo et al., 2019; Elshehabi et al., 2026; Hlavnička et al., 2017; Gunter et al., 2025; Jeancolas et al., 2022; Massimi et al., 2026; Rovini et al., 2018; Schalkamp et al., 2023; Tzfoni et al., 2026; Xie et al., 2026; Prasuhn et al., 2021; Sánchez-Rodríguez et al., 2022; Schalkamp et al., 2025). Reported model performance varied according to study population, digital biomarker domain, analytical method, and validation strategy. Detailed study-level information on digital biomarker features and analytical approaches is provided in Supplementary Table 1.

### 3.4 Evidence characteristics and methodological gaps

The included studies provided several types of evidence, including cross-sectional group differences and discrimination, classification performance, prospective associations with future PD diagnosis or phenoconversion, prospective prediction, and longitudinal monitoring of changes in digital biomarkers. The majority of studies focused on cross-sectional differences between individuals at increased risk of PD and control populations, with alterations reported across gait, upper-limb motor function, speech, sleep, activity, and other digital biomarker domains (Guarín et al., 2025; Arora et al., 2018; Arora et al., 2021; Bertram et al., 2026; Brink-Kjaer et al., 2023; Rusz et al., 2022; Cavallo et al., 2019; Chen et al., 2014; Del Din et al., 2020; Chan et al., 2026; Hlavnička et al., 2017; Gunter et al., 2025; Jeancolas et al., 2022; Illner et al., 2024; Skrabal et al., 2022; Ma et al., 2021; Massimi et al., 2026; Mirelman et al., 2016; Mirelman et al., 2011; Ophey et al., 2025; Rovini et al., 2018; Rusz et al., 2018; Šimek et al., 2026; Simonet et al., 2021; Stefani et al., 2018; Tzfoni et al., 2026; Winer et al., 2023; Zatti et al., 2024; Prasuhn et al., 2021; Sánchez-Rodríguez et al., 2022; Schalkamp et al., 2025). Classification models were also developed using wearable-, smartphone-, or sensor-derived features, although their reported performance varied according to study population, feature set, analytical method, and validation strategy (Guarín et al., 2025; Arora et al., 2018; Arora et al., 2021; Bertram et al., 2026; Brink-Kjaer et al., 2023; Cavallo et al., 2019; Elshehabi et al., 2026; Hlavnička et al., 2017; Gunter et al., 2025; Jeancolas et al., 2022; Illner et al., 2024; Massimi et al., 2026; Rovini et al., 2018; Schalkamp et al., 2023; Tzfoni et al., 2026; Xie et al., 2026; Prasuhn et al., 2021; Sánchez-Rodríguez et al., 2022; Schalkamp et al., 2025). Prospective evidence regarding future PD diagnosis or phenoconversion was available in a smaller subset of studies. Gait-related studies included prospective associations between digital gait features and subsequent phenoconversion or future PD diagnosis, as well as models developed to predict future PD diagnosis or time to diagnosis (Cen et al., 2024; Cen et al., 2026; Del Din et al., 2019; Elshehabi et al., 2026; Pelicioni et al., 2026). Similarly, studies examining activity- and rest-activity patterns included both prospective associations with subsequent PD risk or diagnosis and formal prediction models (Feng et al., 2020; Schalkamp et al., 2023; Winer et al., 2024; Xie et al., 2026). Other longitudinal studies primarily evaluated changes in digital biomarkers over time rather than directly predicting phenoconversion (Bouhadoun et al., 2026; Rusz et al., 2022; Gupta and Patel, 2025; Šimek et al., 2026). Overall, prospective evidence was less extensive than cross-sectional evidence.

Methodological heterogeneity was evident across studies in terms of participant populations, sensor devices, assessment protocols, preprocessing procedures, feature extraction methods, analytical approaches, and validation strategies. Analytical approaches also varied considerably. Random forests, support vector machines, gradient-boosting methods, and neural networks were among the commonly applied machine-learning methods. Validation strategies ranged from conventional cross-validation to nested cross-validation and leave-one-subject-out procedures, whereas independent external validation remained uncommon. Many studies were based on relatively small or single-center cohorts, although multicohort external validation was performed in some studies (Bertram et al., 2026). In addition, studies combining high-dimensional digital features with relatively limited sample sizes raised concerns regarding model overfitting and generalizability (Gunter et al., 2025). Detailed information on the evidence types and validation characteristics of the included studies is provided in Supplementary Table 2. Additional methodological details for studies using machine-learning and AI-based approaches are presented in Supplementary Table 3.

## 4 Discussion

### 4.1 Principal findings

This scoping review mapped the current evidence on digital biomarkers derived from wearable or portable devices for the identification and monitoring of formally defined pPD and populations at increased risk of PD. Across the 43 included studies, digital biomarkers captured a broad spectrum of motor, speech, sleep, activity, and circadian features. Motor-related measures, particularly gait and mobility parameters, represented the most extensively investigated domain. Wearable inertial sensors and smartphone-based assessments were used to quantify subtle motor alterations across different at-risk populations (Cen et al., 2024; Cen et al., 2026; Del Din et al., 2019; Del Din et al., 2020; Elshehabi et al., 2026; Ma et al., 2021; Mirelman et al., 2016; Mirelman et al., 2011; Rovini et al., 2018; Pelicioni et al., 2026; Zatti et al., 2024; Prasuhn et al., 2021; Sánchez-Rodríguez et al., 2022). Speech, sleep, activity, and circadian biomarkers have also received increasing attention, reflecting a broader shift toward multidimensional digital characterization of the prodromal and pre-diagnostic stages of PD (Arora et al., 2021; Bertram et al., 2026; Rusz et al., 2022; Feng et al., 2020; Jeancolas et al., 2022; Illner et al., 2024; Skrabal et al., 2022; Ophey et al., 2025; Schalkamp et al., 2023; Šimek et al., 2026; Stefani et al., 2018; Tzfoni et al., 2026; Winer et al., 2023; Winer et al., 2024; Xie et al., 2026; Schalkamp et al., 2025).

The strength and maturity of evidence, however, varied considerably across applications. The majority of studies provided cross-sectional evidence of group differences or discrimination, whereas prospective evidence regarding future PD diagnosis or phenoconversion was less extensive and included both longitudinal associations and formal prediction models (Cen et al., 2024; Cen et al., 2026; Del Din et al., 2019; Elshehabi et al., 2026; Feng et al., 2020; Pelicioni et al., 2026; Schalkamp et al., 2023; Winer et al., 2024; Xie et al., 2026). Other longitudinal studies primarily evaluated changes in digital biomarkers over time rather than directly predicting phenoconversion (Bouhadoun et al., 2026; Rusz et al., 2022; Gupta and Patel, 2025; Šimek et al., 2026). Multimodal and AI-based approaches further expanded the ability to integrate and analyze complex digital features. However, their potential for clinical translation remains constrained by heterogeneity in study populations, measurement protocols, analytical approaches, and validation strategies. Overall, wearable- and portable-device-derived measures represent promising candidate biomarkers for characterizing and monitoring individuals across the prodromal PD risk spectrum. Nevertheless, robust evidence supporting their use for clinical risk stratification and the prediction of phenoconversion remains limited. Previous reviews have examined digital biomarkers across the broader spectrum of PD diagnosis and monitoring (Sun et al., 2024). In contrast, the present review specifically focuses on the prodromal PD risk spectrum and distinguishes cross-sectional evidence of discrimination and association from longitudinal monitoring and prospective evidence of phenoconversion.

### 4.2 From motor abnormalities to multidimensional digital phenotyping of prodromal PD

Motor-related digital biomarkers represented the most extensively investigated domain in this review, reflecting both the clinical characteristics of PD and the relative maturity of technologies for objectively quantifying motor abnormalities. Gait, mobility, upper-limb function, and postural control can be assessed using wearable inertial sensors, accelerometers, and smartphone-based platforms, providing quantitative measures beyond those obtained during episodic clinical assessments. Among the included studies, gait-related parameters, including stride characteristics, variability, asymmetry, turning, and mobility patterns, were frequently investigated in individuals at increased risk of PD (Cen et al., 2024; Cen et al., 2026; Del Din et al., 2019; Del Din et al., 2020; Elshehabi et al., 2026; Ma et al., 2021; Mirelman et al., 2016; Mirelman et al., 2011; Rovini et al., 2018; Pelicioni et al., 2026; Zatti et al., 2024; Prasuhn et al., 2021; Sánchez-Rodríguez et al., 2022). This emphasis is consistent with previous evidence indicating that subtle motor abnormalities may emerge during the prodromal phase and that quantitative motor assessments may help characterize motor alterations associated with prodromal PD (Simonet et al., 2021; Heinzel et al., 2019).

However, the prodromal and pre-diagnostic phases of PD are characterized by heterogeneous motor and non-motor manifestations. Established prodromal markers, including REM sleep behavior disorder, olfactory impairment, autonomic dysfunction, and neuropsychiatric symptoms, indicate that disease-related alterations may involve multiple systems before clinical diagnosis (Heinzel et al., 2019; Postuma and Berg, 2016). Consistent with this concept, our review identified digital biomarkers derived from speech, sleep, physical activity, and circadian rhythms, in addition to motor measures (Arora et al., 2021; Bertram et al., 2026; Brink-Kjaer et al., 2023; Rusz et al., 2022; Chan et al., 2026; Feng et al., 2020; Hlavnička et al., 2017; Jeancolas et al., 2022; Illner et al., 2024; Skrabal et al., 2022; Massimi et al., 2026; Ophey et al., 2025; Rusz et al., 2018; Schalkamp et al., 2023; Šimek et al., 2026; Stefani et al., 2018; Tzfoni et al., 2026; Winer et al., 2023; Winer et al., 2024; Xie et al., 2026; Schalkamp et al., 2025). These measures may provide complementary information by capturing behavioral and physiological alterations that are difficult to characterize through conventional episodic clinical assessments. Nevertheless, compared with motor measures, the evidence supporting non-motor digital biomarkers remains less established, particularly with respect to disease-specific validation, sensitivity to longitudinal change, and external validation (Janssen Daalen et al., 2024).

The extension from single-domain measurements to multidimensional digital phenotyping may therefore represent an important development in research across the prodromal PD risk spectrum. Individual digital features capture only specific aspects of a heterogeneous disease process and may also be influenced by aging, comorbidities, environmental factors, and interindividual variability. Integrating complementary information across motor, speech, sleep, and activity domains could provide a more comprehensive digital representation of individuals across the prodromal PD risk spectrum. However, evidence that multidimensional profiles provide incremental predictive value beyond individual digital features or established clinical and biological risk markers remains limited. Establishing this added value will be important before multidimensional digital phenotyping can contribute meaningfully to clinical risk stratification and the prediction of phenoconversion.

### 4.3 Multimodal integration and AI-based approaches: promise and challenges

The increasing availability of wearable and portable devices has created opportunities to move beyond single-domain measurements toward multidimensional digital phenotyping across the prodromal PD risk spectrum. In this review, a small number of studies integrated information across distinct functional domains, including motor performance, speech, balance, dexterity, and other behavioral features (Arora et al., 2018; Bouhadoun et al., 2026; Gunter et al., 2025). Such integration is conceptually relevant because the heterogeneous manifestations of prodromal PD are unlikely to be fully captured by any single digital feature or functional domain (Sun et al., 2024; Berg et al., 2021). By combining complementary information across motor and non-motor domains, multimodal approaches may provide a more comprehensive representation of individuals across the prodromal PD risk spectrum. They may also capture complementary abnormalities that emerge at different stages or reflect different components of the prodromal disease process, potentially reducing reliance on any single feature that may be influenced by aging, comorbidities, or interindividual variability. However, given the small number of genuinely multidomain studies identified in this review, evidence that multimodal models provide incremental predictive value beyond individual digital biomarkers or established clinical and biological risk markers remains limited. Their superiority over single-domain approaches has therefore not yet been established.

Machine-learning and AI-based methods offer analytical tools for extracting and integrating complex patterns from high-dimensional sensor-derived data. These methods were applied across motor, speech, sleep/activity, and multidomain studies for digital feature extraction, classification, and prediction (Guarín et al., 2025; Arora et al., 2018; Arora et al., 2021; Bertram et al., 2026; Brink-Kjaer et al., 2023; Cavallo et al., 2019; Elshehabi et al., 2026; Hlavnička et al., 2017; Gunter et al., 2025; Jeancolas et al., 2022; Massimi et al., 2026; Rovini et al., 2018; Schalkamp et al., 2023; Tzfoni et al., 2026; Xie et al., 2026; Prasuhn et al., 2021; Sánchez-Rodríguez et al., 2022; Schalkamp et al., 2025). Although these approaches may facilitate the detection of subtle and non-linear patterns, promising model performance should be interpreted cautiously. High feature dimensionality relative to sample size may increase the risk of overfitting, whereas limited independent external validation may restrict model generalizability. Potential risks related to data leakage, class imbalance, and limited interpretability should also be considered during model development and evaluation (Collins et al., 2024; Moons et al., 2025). Moreover, reported performance metrics were derived from heterogeneous populations, tasks, feature sets, and validation strategies and should therefore not be interpreted as directly comparable measures of model performance across studies. Accordingly, the value of AI-based digital biomarkers should not be assessed solely on the basis of classification performance. Future research should establish whether these models provide reproducible and clinically meaningful incremental value, particularly for predicting phenoconversion rather than merely distinguishing at-risk groups from control populations.

### 4.4 Translating digital biomarkers from association to clinical prediction

A major challenge in the field of prodromal PD digital biomarkers is the transition from detecting disease-associated abnormalities to predicting future clinical conversion. Although numerous studies have demonstrated that wearable- and portable-device-derived measures can identify subtle differences between individuals at increased risk of PD and control populations, such findings primarily indicate the presence of altered digital phenotypes rather than demonstrating the ability to predict which individuals will subsequently develop clinically diagnosed PD. This distinction is particularly important because high-risk populations, including individuals with iRBD, hyposmia, genetic susceptibility, or other prodromal features, are heterogeneous in both their probability and time to phenoconversion (Heinzel et al., 2019; Postuma and Berg, 2016). In this review, prospective evidence was considerably less extensive than cross-sectional evidence. Prospective gait-related evidence included associations of stride asymmetry and other gait features with subsequent phenoconversion or future PD diagnosis, as well as studies that developed models to predict future PD diagnosis or time to diagnosis (Cen et al., 2024; Cen et al., 2026; Del Din et al., 2019; Elshehabi et al., 2026; Pelicioni et al., 2026). Similarly, studies examining activity- and rest-activity patterns included both prospective associations with subsequent PD risk or diagnosis and formal prediction models (Feng et al., 2020; Schalkamp et al., 2023; Winer et al., 2024; Xie et al., 2026). Longitudinal monitoring evidence was also reported for digital bradykinesia and speech biomarkers, although such evidence remained limited overall (Bouhadoun et al., 2026; Rusz et al., 2022; Šimek et al., 2026). Collectively, these findings suggest that digital biomarkers may capture evolving changes during the pre-diagnostic phase. However, their incremental predictive value beyond established clinical and biological risk markers remains uncertain.

Translating digital biomarkers into clinically useful prediction tools will therefore require a shift from cross-sectional discrimination and exploratory biomarker discovery toward rigorous longitudinal validation. Future studies should prioritize large, multicenter cohorts with standardized digital assessment protocols, repeated measurements, clearly defined phenoconversion outcomes, and independent external validation. Digital biomarkers may also have greater clinical value when integrated with complementary clinical, genetic, imaging, and biological information rather than considered as standalone predictors (Yu et al., 2026). Rather than replacing established prodromal PD criteria, digital biomarkers may complement existing risk frameworks by providing objective and repeated measures of motor and non-motor changes over time. Future studies should determine whether incorporating digital measures into established frameworks, such as the MDS research criteria for prodromal PD (Berg et al., 2015), improves individualized risk estimation and the prediction of phenoconversion. However, the added predictive value and clinical utility of such integration must be established before implementation in clinical practice.

### 4.5 Limitations and future directions

This scoping review has several limitations. First, substantial heterogeneity in study populations, device types, assessment protocols, digital features, analytical methods, and reported outcomes precluded the quantitative synthesis of classification and predictive performance. Second, consistent with the objectives of a scoping review, this review did not undertake a formal assessment of methodological quality or risk of bias. Therefore, the reported performance metrics and associations should be interpreted in light of the methodological limitations of the individual studies. Third, only English-language peer-reviewed journal articles were included, which may have resulted in the omission of relevant evidence published in other languages or in non-peer-reviewed sources. From an engineering and signal-processing perspective, differences in sensor characteristics, data acquisition protocols, preprocessing procedures, and feature extraction methods may affect the reproducibility and comparability of digital biomarkers across devices and studies. Future research should prioritize standardized data acquisition and preprocessing, consistent feature definitions and analytical procedures, and transparent reporting to improve reproducibility and comparability across studies (Badier et al., 2026). Large, multicenter longitudinal cohorts with repeated assessments, clearly defined phenoconversion outcomes, and independent external validation are needed to establish the predictive value and generalizability of digital biomarkers (Janssen Daalen et al., 2024). Independent external validation should be prioritized, particularly for high-dimensional machine-learning and AI-based models, to establish their robustness and generalizability across populations, devices, and assessment settings. Beyond methodological validation, future studies should also address data privacy, governance, accessibility, and digital equity, as well as practical deployment challenges, including battery life, data-transmission reliability, long-term user adherence, and the burden of repeated active smartphone-based tasks, particularly among older populations. These factors may influence the feasibility and scalability of wearable- and portable-device-derived assessment in clinical and community settings (Capulli et al., 2025).

## 5 Conclusion

This scoping review provides a comprehensive overview of digital biomarkers derived from wearable and portable devices for the identification and monitoring of formally defined pPD and populations at increased risk of PD. Motor-related biomarkers, particularly gait and mobility measures, represented the most extensively investigated domain, whereas speech, sleep, activity, and multidomain digital features provided complementary approaches to characterizing the heterogeneous manifestations of the prodromal and pre-diagnostic phases of PD. Multimodal integration and AI-based approaches offer potential for multidimensional characterization. However, current evidence remains constrained by methodological heterogeneity and limited longitudinal and external validation. Therefore, wearable- and portable-device-derived digital biomarkers should currently be considered promising candidate tools rather than established approaches for clinical prediction. Future research should prioritize standardized assessment, longitudinal and external validation, and integration with established clinical and biological markers to determine the value of these biomarkers for individualized PD risk assessment.
