# Supplementary material for: Digital biomarkers derived from wearable and portable devices in prodromal Parkinson’s disease and at-risk populations: a scoping review
Source: Front Aging Neurosci. 2026 Oct 12;18:1850991. doi: 10.3389/fnagi.2026.1850991 (PMC13697243; doi:10.3389/fnagi.2026.1850991)
Supplement: Supplementary file 1 [file Data_Sheet_1.docx]

Appendix 1	Search strategies

| Data sources | Search | Query | Results |
| --- | --- | --- | --- |
| Pubmed | **#1** | "Parkinson Disease"[MeSH Terms] OR "parkinson*"[Title/Abstract] | 187,384 |
|  | **#2** | "Prodromal Symptoms"[MeSH Terms] OR "prodromal"[Title/Abstract] OR "premotor"[Title/Abstract] OR "preclinical"[Title/Abstract] OR "pre-clinical"[Title/Abstract] OR "premanifest"[Title/Abstract] OR "pre-manifest"[Title/Abstract] OR "at risk"[Title/Abstract] OR "high risk"[Title/Abstract] OR "rem sleep behavior disorder"[MeSH Terms] OR "rem sleep behavior disorder"[Title/Abstract] OR "REM sleep behaviour disorder"[Title/Abstract] OR "iRBD"[Title/Abstract] OR "Olfaction Disorders"[MeSH Terms] OR "hyposmia"[Title/Abstract] OR "anosmia"[Title/Abstract] OR "olfactory"[Title/Abstract] OR "non-manifesting"[Title/Abstract] OR "nonmanifesting"[Title/Abstract] OR "mutation carrier*"[Title/Abstract] OR "LRRK2"[Title/Abstract] OR "PRKN"[Title/Abstract] OR "Parkin"[Title/Abstract] OR "GBA"[Title/Abstract] | 1,034,833 |
|  | **#3** | "Wearable Electronic Devices"[MeSH Terms] OR "wearable*"[Title/Abstract] OR "inertial"[Title/Abstract] OR "acceleromet*"[Title/Abstract] OR "IMU"[Title/Abstract] OR "gyroscope*"[Title/Abstract] OR "sensor*"[Title/Abstract] OR "actigraph*"[Title/Abstract] OR "smartwatch*"[Title/Abstract] OR "smartphone*"[Title/Abstract] OR "wristband*"[Title/Abstract] OR "smart bracelet*"[Title/Abstract] OR "portable"[Title/Abstract] OR "touchscreen*"[Title/Abstract] OR "microphone*"[Title/Abstract] OR "camera*"[Title/Abstract] OR "video-based"[Title/Abstract] | 816,027 |
|  | **#4** | #1 AND #2 AND #3 | 769 |
| Web of Science | **#1** | TS=("Parkinson Disease" OR parkinson*) | 256,397 |
|  | **#2** | TS=("Prodromal Symptoms" OR prodromal OR premotor OR preclinical OR "pre-clinical" OR premanifest OR "pre-manifest" OR "at risk" OR "high risk" OR "REM Sleep Behavior Disorder" OR "REM sleep behaviour disorder" OR iRBD OR "Olfaction Disorders" OR hyposmia OR anosmia OR olfactory OR "non-manifesting" OR nonmanifesting OR "mutation carrier*" OR LRRK2 OR PRKN OR Parkin OR GBA) | 1,148,947 |
|  | **#3** | TS=( "Wearable Electronic Devices" OR wearable* OR inertial OR acceleromet* OR IMU OR gyroscope* OR sensor* OR actigraph* OR smartwatch* OR smartphone* OR wristband* OR "smart bracelet*" OR portable OR touchscreen* OR microphone* OR camera* OR video-based) | 2,505,038 |
|  | **#4** | #1 AND #2 AND #3 | 1,152 |
| **Embase** | **#1** | 'parkinson disease':ab,ti OR parkinson*:ab,ti | 260,723 |
|  | **#2** | 'prodromal symptoms':ab,ti OR prodromal:ab,ti OR premotor:ab,ti OR preclinical:ab,ti OR 'pre-clinical':ab,ti OR premanifest:ab,ti OR 'pre-manifest':ab,ti OR 'at risk':ab,ti OR 'high risk':ab,ti OR 'rem sleep behavior disorder':ab,ti OR 'rem sleep behaviour disorder':ab,ti OR irbd:ab,ti OR 'olfaction disorders':ab,ti OR hyposmia:ab,ti OR anosmia:ab,ti OR olfactory:ab,ti OR 'non-manifesting':ab,ti OR nonmanifesting:ab,ti OR 'mutation carrier*':ab,ti OR lrrk2:ab,ti OR prkn:ab,ti OR parkin:ab,ti OR gba:ab,ti | 1,597,532 |
|  | **#3** | 'wearable electronic devices' OR wearable* OR inertial OR acceleromet* OR imu OR gyroscope* OR sensor* OR actigraph* OR smartwatch* OR smartphone* OR wristband* OR 'smart bracelet*' OR portable OR touchscreen* OR microphone* OR camera* OR 'video based education' | 1,276,987 |
|  | **#4** | #1 AND #2 AND #3 | 1,712 |
| Scopus | **#1** | TITLE-ABS-KEY ( "Parkinson's disease" OR parkinson* ) | 273,811 |
|  | **#2** | TITLE-ABS-KEY ( "Prodromal Symptoms" OR prodromal OR premotor OR preclinical OR "pre-clinical" OR premanifest OR "pre-manifest" OR "at risk" OR "high risk" OR "REM Sleep Behavior Disorder" OR "REM sleep behaviour disorder" OR iRBD OR "Olfaction Disorders" OR hyposmia OR anosmia OR olfactory OR "non-manifesting" OR nonmanifesting OR "mutation carrier*" OR LRRK2 OR PRKN OR Parkin OR GBA ) | 1,612,233 |
|  | **#3** | TITLE-ABS-KEY ( "wearable electronic devices" OR wearable* OR inertial OR acceleromet* OR IMU OR gyroscope* OR sensor* OR actigraph* OR smartwatch* OR smartphone* OR wristband* OR "smart bracelet*" OR portable OR touchscreen* OR microphone* OR camera* OR "video-based" ) | 3,618,184 |
|  | **#4** | #1 AND #2 AND #3 | 1,544 |
| IEEE Xplore | **#1** | "All Metadata":"Parkinson disease" OR "All Metadata":parkinson* | 6,757 |
|  | **#2** | ("All Metadata":"Prodromal Symptoms" OR "All Metadata":prodromal OR "All Metadata":premotor OR "All Metadata":preclinical OR "All Metadata":"pre-clinical" OR "All Metadata":premanifest OR "All Metadata":"pre-manifest" OR "All Metadata":"at risk" OR "All Metadata":"high risk" OR "All Metadata":"REM Sleep Behavior Disorder" OR "All Metadata":"REM sleep behaviour disorder" OR "All Metadata":iRBD OR "All Metadata":"Olfaction Disorders" OR "All Metadata":hyposmia OR "All Metadata":anosmia OR "All Metadata":olfactory OR "All Metadata":"non-manifesting" OR "All Metadata":nonmanifesting OR "All Metadata":"mutation carrier*" OR "All Metadata":LRRK2 OR "All Metadata":PRKN OR "All Metadata":Parkin OR "All Metadata":GBA) | 26,125 |
|  | **#3** | ("All Metadata":"wearable electronic devices" OR "All Metadata":wearable OR "All Metadata":wearables OR "All Metadata":inertial OR "All Metadata":accelerometer OR "All Metadata":accelerometers OR "All Metadata":IMU OR "All Metadata":gyroscope OR "All Metadata":gyroscopes OR "All Metadata":sensor OR "All Metadata":sensors OR "All Metadata":actigraph OR "All Metadata":actigraphy OR "All Metadata":smartwatch OR "All Metadata":smartwatches OR "All Metadata":smartphone OR "All Metadata":smartphones OR "All Metadata":wristband OR "All Metadata":wristbands OR "All Metadata":"smart bracelet" OR "All Metadata":"smart bracelets" OR "All Metadata":portable OR "All Metadata":touchscreen OR "All Metadata":touchscreens OR "All Metadata":microphone OR "All Metadata":microphones OR "All Metadata":camera OR "All Metadata":cameras OR "All Metadata":"video-based") | 1,172,459 |
|  | **#4** | #1 AND #2 AND #3 | 68 |
| ACM Digital Library | **#1** | [Abstract: "parkinson disease"] OR [Abstract: parkinson*] | 2,172 |
|  | **#2** | [Abstract: "prodromal symptoms"] OR [Abstract: prodromal] OR [Abstract: premotor] OR [Abstract: preclinical] OR [Abstract: "pre-clinical"] OR [Abstract: premanifest] OR [Abstract: "pre-manifest"] OR [Abstract: "at risk"] OR [Abstract: "high risk"] OR [Abstract: "rem sleep behavior disorder"] OR [Abstract: "rem sleep behaviour disorder"] OR [Abstract: irbd] OR [Abstract: "olfaction disorders"] OR [Abstract: hyposmia] OR [Abstract: anosmia] OR [Abstract: olfactory] OR [Abstract: "non-manifesting"] OR [Abstract: nonmanifesting] OR [Abstract: "mutation carrier*"] OR [Abstract: lrrk2] OR [Abstract: prkn] OR [Abstract: parkin] OR [Abstract: gba] | 10,527 |
|  | **#3** | [Abstract: wearable] OR [Abstract: wearables] OR [Abstract: inertial] OR [Abstract: accelerometer] OR [Abstract: accelerometers] OR [Abstract: imu] OR [Abstract: gyroscope] OR [Abstract: gyroscopes] OR [Abstract: sensor] OR [Abstract: sensors] OR [Abstract: sensing] OR [Abstract: actigraph] OR [Abstract: actigraphy] OR [Abstract: smartwatch] OR [Abstract: smartwatches] OR [Abstract: smartphone] OR [Abstract: smartphones] OR [Abstract: "mobile phone"] OR [Abstract: "mobile phones"] OR [Abstract: "mobile device"] OR [Abstract: "mobile devices"] OR [Abstract: wristband] OR [Abstract: wristbands] OR [Abstract: "smart bracelet"] OR [Abstract: "smart bracelets"] OR [Abstract: portable] OR [Abstract: touchscreen] OR [Abstract: touchscreens] OR [Abstract: microphone] OR [Abstract: microphones] OR [Abstract: camera] OR [Abstract: cameras] OR [Abstract: video] OR [Abstract: "video-based"] | 710,663 |
|  | **#4** | #1 AND #2 AND #3 | 34 |

# **Supplementary Table 1. Digital Biomarkers and Analytical Approaches**

| **Author / Year** | **Biomarker Domain** | **Key Features** | **Feature Extraction** | **Analysis** |
| --- | --- | --- | --- | --- |
| Guarín et al., 2025 (11) | Upper-limb/fine motor | Amplitude; speed; amplitude & speed decrement | Video-based hand-pose tracking | ANCOVA; LR/SVM/NB/DT/RF |
| Arora et al., 2018  (12) | Multidomain | Voice, balance, gait, tapping, reaction time, rest & postural tremor features | Task-specific smartphone feature extraction; 998 features → top 30 | RF; feature ranking; CV |
| Arora et al., 2021  (13) | Speech/voice | 337 acoustic features: jitter, shimmer, MFCCs, noise & nonlinear measures | Automated segmentation + acoustic signal processing | RF; Spearman correlation |
| Bertram et al., 2026  (14) | Sleep/nocturnal motor activity | Movement intensity, periodicity, spectral properties, complexity, fragmentation, activity level & clustering | Automated sleep segmentation + activity-bout detection; global/local handcrafted motion features | XGBoost; nested CV; external validation; LODO-CV |
| Bouhadoun et al., 2026 (15) | Multidomain | Bradykinesia and speech summary scores; hand-turning amplitude; U-turn step-time variability; speech level/CPP | Pre-specified smartphone-derived features aggregated over 2-week periods | ANCOVA; LME/MMRM; logistic regression |
| Brink-Kjaer et al., 2023 (16) | Sleep/nocturnal motor activity | Activity index; SIB; twitch activity; long immobile bouts; Hjorth parameters | High-frequency activity-count features; probable-REM time-window analysis | Ensemble decision trees; SVM; logistic regression |
| Rusz et al., 2022  (17) | Speech | Fundamental frequency variability (F0SD); composite F0SD | Automated acoustic analysis of reading and monologue | ANCOVA; paired longitudinal analysis |
| Cavallo et al., 2019  (18) | Upper-limb motor function | Velocity, amplitude, frequency, energy, movement variability | Spatiotemporal & frequency-domain inertial analysis | SVM; RF; Naïve Bayes |
| Cen et al., 2024  (19) | Gait | Stride length/time/velocity; variability; asymmetry; arm swing; trunk motion | Sensor-derived spatiotemporal gait parameters grouped into gait domains | Repeated-measures ANOVA; Cox regression; Spearman correlation |
| Cen et al., 2026  (20) | Gait | Stride length/velocity/time; variability; asymmetry; arm swing; trunk motion; dual-task cost | Automated spatiotemporal gait analysis | Cox regression; competing-risk regression; GEE |
| Chen et al., 2014  (21) | Balance/postural sway | RMS acceleration; JERK; AP/ML sway components | Filtered tri-axial acceleration; automated sway-parameter extraction | Repeated-measures ANOVA; post-hoc t-tests; Spearman correlation |
| Del Din et al., 2019  (22) | Gait | 14 gait characteristics across pace, variability, rhythm, asymmetry, and postural control | Validated accelerometer-based gait algorithm; gait-event detection and derivation of spatiotemporal features | Cox regression; forward/backward stepwise Cox models; random-effects linear mixed models |
| Del Din et al., 2020  (23) | Gait | Macro: walking volume, pattern, variability; Micro: pace, variability, rhythm, asymmetry, postural control | Automated free-living walking-bout detection; 14 Micro gait characteristics stratified by bout duration | Group comparisons; ROC analysis; binary logistic regression; partial correlations |
| Elshehabi et al., 2026 (24) | Gait/turning | Turn angle; turn duration; start/middle/end, mean & peak angular velocity | Validated algorithm deriving 7 turning measures from accelerometer/gyroscope signals | Linear mixed-effects models; Cox regression; XGBoost classification |
| Chan et al., 2026  (25) | Pupillary response / circadian activity | Net PIPR-6s; PIPR-6s; RAR amplitude, mesor, acrophase, IS, IV, M10; sleep efficiency, WASO | Blue/red light–evoked pupillary response; NPCRA and cosinor analysis of wrist actigraphy | Ordinal logistic regression; generalized linear models; linear regression |
| Feng et al., 2020  (26) | Rest–activity / sleep–circadian / physical activity | Probable napping %, duration & episodes; kRA/kAR; average weekly activity; amplitude; acrophase; IS; IV; L5/L5o; M10/M10o; active-period activity | 1-min epochs; sleep/nap detection; state-transition analysis; cosinor and nonparametric rest–activity analysis | ANCOVA/nonparametric tests; FDR & Bonferroni correction; functional linear modeling; matched analyses |
| Hlavnička et al., 2017 (27) | Speech | 12 acoustic features covering timing, articulation, phonation and respiration | Fully automated segmentation of voiced/unvoiced speech, pauses and respiration; GMM-based signal processing | RM-ANOVA; quadratic discriminant analysis; leave-one-subject-out CV |
| Gunter et al., 2025  (28) | Multidomain | Gait, tremor, voice, dexterity, balance and reaction-time features | 1,058 smartphone-derived features from accelerometer, microphone and touchscreen data | XGBoost; logistic regression; SHAP; nested/repeated 5-fold CV; XGBoost regression |
| Gupta & Patel, 2025  (29) | Free-living motor activity | Submovement distance, peak velocity, acceleration, variability; activity intensity | Automated extraction of 85 wrist-movement features, including 24 submovement kinematic measures | LASSO regression; pairwise logistic model; 5-fold CV; longitudinal slope analysis |
| Jeancolas et al., 2022 (30) | Speech | Prosody, pause duration, rhythm, phonation and low-level spectral/articulatory features | Automated acoustic extraction using PRAAT/Matlab; high-level features plus MFCC-GMM and x-vector representations | MANCOVA/ANOVA; SVM; MFCC-GMM; x-vector classifiers; majority-vote fusion |
| Illner et al., 2024  (31) | Speech | Monopitch, imprecise vowel articulation, voice quality, articulatory decay, monoloudness, prolonged pauses, articulation rate | Automated extraction of 7 predefined acoustic features from passive calls and active reading; MATLAB/Python; call segmentation and preprocessing | ANOVA/Bonferroni; Spearman correlation; binary logistic regression; LOOCV; ROC/AUC; exhaustive feature-combination search |
| Skrabal et al., 2022  (32) | Speech | Vowel Space Area (VSA); Vowel Articulation Index (VAI); vowel duration; F1/F2 formant frequencies | PRAAT-based acoustic analysis of 30 corner vowels (/a/, /i/, /u/); F1/F2 extraction and calculation of VSA/VAI | ANCOVA with Bonferroni correction; age-adjusted partial Pearson correlations |
| Ma et al., 2021  (33) | Gait | Stride length/velocity/time; stride length asymmetry; stride length/time variability; trunk range of motion and peak angular velocity; step time before turning | APDM-derived spatiotemporal gait and trunk-motion parameters across usual, fast and dual-task walking | Repeated-measures ANOVA; ANCOVA; Bonferroni post hoc tests; Pearson correlations |
| Maremmani et al., 2018 (34) | Upper- and lower-limb motor function | THMF; OPCL; PSUP; TTFCV; 1-year percentage change in motor performance | Sensor-derived movement counts/frequency variability; 3 repetitions/task averaged; longitudinal percentage change from baseline | Within-subject longitudinal change; predefined >15% deterioration threshold; t test/Mann–Whitney U; Pearson test–retest correlation |
| Massimi et al., 2026  (35) | Sleep / nocturnal motor activity | Mean motor activity (MA); activity index (AI); short/long immobile bouts (SIB/LIB); twitch activity (TA); sleep-related movement features | Automated wear/sleep detection; 1-s activity counts; 113 candidate features derived from four primary movement constructs and multiple time windows | XGBoost; fully nested stratified group CV; Bayesian hyperparameter optimization; subject-level probability aggregation |
| Mirelman et al., 2016 (36) | Gait / arm swing | Arm swing amplitude, asymmetry, variability and jerk; axial rotation amplitude, asymmetry and jerk; gait speed; stride-time variability | Synchronized wrist/lower-back sensor signals; straight-line walking isolated; sensor-derived arm-swing, axial-rotation and gait parameters | Linear mixed models with covariate adjustment and Bonferroni correction; Pearson correlations; linear regression |
| Mirelman et al., 2011 (37) | Gait | Gait speed; stride time; stride length; stride-time variability; dominant-frequency peak amplitude | Lower-back accelerometry; stride-time CV; spectral analysis of calibrated acceleration signal in 0.5–3.0 Hz locomotion band | Repeated-measures ANOVA; post-hoc Student’s t-tests; Bonferroni correction |
| Ophey et al., 2025  (38) | Physical activity / Sleep / Circadian rhythm | Total-ENMO; inactivity, LPA, MPA, VPA; sleep duration, sleep efficiency, WASO; SRI, IS, IV | GGIR v3.1.4; auto-calibration; wear-time QC; ENMO activity thresholds; HDCZA sleep detection; non-parametric circadian metrics | Mann–Whitney U; age-adjusted partial Spearman correlations with FDR correction; elastic-net regression with nested 10-fold CV |
| Rovini et al., 2018  (39) | Lower-limb motor function / Gait | Gait time, number of strides, gait/rotation frequency, stride/swing/stance time, relative stance, foot angular excursion, tapping frequency/number/amplitude variability, and spectral/energy features | Butterworth filtering; task-specific signal segmentation; angular-rate integration and drift correction; 23 features per limb extracted; significance testing followed by removal of highly correlated features (ρ > 0.85) | Kruskal–Wallis / Wilcoxon rank-sum tests; Spearman correlation; SVM, Random Forest, and Naïve Bayes; 10-fold CV |
| Rusz et al., 2018  (40) | Speech / Voice | F0 SD; duration of pause intervals (DPI); rate of speech timing (RST); additionally assessed jitter, shimmer, HNR, DDK rate/regularity, VOT, Int SD, and RFA | One-way ANOVA with post-hoc testing; Pearson correlation and NRMSE; binary logistic regression with LOOCV; ROC/AUC; post-hoc RBD-pPD vs RBD-A comparison | One-way ANOVA with post-hoc testing; Pearson correlation and NRMSE; binary logistic regression with LOOCV; ROC/AUC; post-hoc RBD-pPD vs RBD-A comparison |
| Pelicioni et al., 2026  (41) | Gait | Daily step count; running duration; usual/maximum walking speed; cadence; step-time variability; step/stride regularity; walking-bout duration/distribution; natural arm swing and static/adaptive hand positions | Watch Walk v2.3 framework; 17 digital gait biomarkers derived from wrist accelerometry using signal-processing techniques and machine-learning regression/classification models | Age- and sex-adjusted ANOVA with Bonferroni post-hoc tests; Cohen’s d; age- and sex-adjusted Cox regression; multivariable Cox models |
| Schalkamp et al., 2023 (42) | Physical activity / sleep | Average acceleration; activity-class movement and duration; interruptions; light activity; MVPA; sleep duration/continuity; nocturnal awakenings; daytime naps | Raw accelerometry preprocessing; 30-s epoch classification into sleep, sedentary, light and MVPA; derived activity/sleep phenotypes | Lasso logistic regression; nested 5-fold CV; survival random forest; time-dependent AUROC; AUPRC; calibration analysis |
| Šimek et al., 2026  (43) | Speech / Voice | F0 SD (monopitch); FRI (vowel articulation); CPP (voice quality); NSR (articulation rate); LTAS mean; global MFCC; neural embedding index | Silence >0.7 s removed; recordings segmented into 20-s windows; segment-level features aggregated by median; 4 predefined acoustic features plus 3 global speech-impairment descriptors; neural embedding index derived from pretrained TRILL-distilled 2048-dimensional embeddings | Linear mixed-effects models for longitudinal progression; Huber regression for individual slopes; age-adjusted partial Pearson correlations; time-to-event and clinical-trial sample-size analyses |
| Simonett et al., 2021 (44) | Upper-limb motor function / Bradykinesia | Finger tapping amplitude; velocity; frequency; slope; coefficient of variation (CV); velocity-frequency combination parameters | Smartphone video processed using convolutional neural network (CNN) hand landmark detection; tracking thumb and index finger landmarks; extraction of movement kinematics | Logistic regression; ROC analysis; AUC, sensitivity, specificity; correlation with MDS-UPDRS III finger tapping scores |
| Stefani et al., 2018  (45) | Sleep-related motor activity / nocturnal movement | Activity score; activity index; acceleration index; brief wake ratio; short burst inactivity index; probable wakefulness episodes | Actigraphy data processed using Motionlogger WatchWare and Action-W software; nocturnal rest period manually determined; activity-related epochs extracted | Quantitative group comparison; blinded expert visual classification of actigraphy patterns; ROC analysis; sensitivity, specificity, accuracy, AUC; inter-rater reliability |
| Tzfoni et al., 2026  (46) | Sleep-related nocturnal mobility / motor activity | Activity count; axis-specific acceleration features; immobile bouts; twitch activity; Hjorth parameters; sleep-related mobility metrics | 126 features extracted from acceleration signals; after removing highly correlated features, 112 retained | PCA exploration; SHAP interpretation; feature selection (RFE); ML classifiers (SVM, Logistic Regression, Random Forest, Gradient Boosting); LOOCV |
| Winer et al., 2023  (47) | Sleep-wake rhythm / Circadian activity biomarkers | Cosinor amplitude, mesor; intradaily variability (IV); interdaily stability (IS); least active 5 h (L5); most active 10 h (M10); fPCA-derived activity patterns | Raw tri-axial accelerometer data processed into 30-s epochs; non-wear correction; calculation of 24-h rest–activity rhythm parameters | Cosinor analysis; non-parametric actigraphy analysis; functional principal component analysis (fPCA); Mann–Whitney U tests |
| Zatti et al., 2024  (48) | Gait / Turning mobility biomarkers | Turning duration; mean angular velocity; peak angular velocity | Lower-back IMU placed at L5 level; raw accelerometer and gyroscope signals segmented into TUG walking trials; turning detected using validated algorithm based on 6DOF attitude estimation and yaw angular displacement | ANCOVA adjusted for age and sex; Bonferroni correction; between-group comparison of digital turning parameters |
| Winer et al., 2024  (49) | Activity rhythm / Circadian rest-activity biomarkers | Mesor; amplitude; interdaily stability (IS); intradaily variability (IV); least active 5 h (L5); most active 10 h (M10); fPCA components | Wrist accelerometer data processed into 30-s epochs; cosinor analysis, nonparametric rhythm analysis and functional principal component analysis (fPCA) | Cox proportional hazards models for incident PD/AD; linear mixed models for cognitive decline |
| Xie et al., 2026  (50) | Sleep architecture + physical activity dynamics | Raw triaxial accelerometry | Pre-trained activity recognition model + self-supervised sleep staging model + Markov transition features | XGBoost-Cox survival model |
| Prasuhn et al., 2021  (51) | Gait and postural control motor biomarkers | Postural sway features; gait-related motion metrics; dual-task gait parameters | Raw IMU signals processed into interpretable mobility metrics during different gait and posturography conditions | Gradient boosted trees (GBT) classification models; balanced accuracy; ROC-AUC and other classification metrics |
| Sanchez-Rodríguez et al., 2022 (52) | Gait motor biomarkers | Gait speed; step/stride time; step length; arm swing amplitude; variability; asymmetry; turning parameters | Sensor-recorded quaternion data converted into joint angles and spatiotemporal gait variables | Neural network classification; statistical comparison; correlation with DaT-SPECT |
| Schalkamp et al., 2025 (53) | Physical activity, sleep, and vital signs digital biomarkers | Step count, walking minutes, sleep duration, REM/NREM sleep, sleep efficiency, awakenings, pulse rate, RMSSD | Verily-derived hourly time-series features; tsfresh extracted temporal characteristics (maximum, minimum, skewness, kurtosis, trend) | Elastic-net logistic regression model to generate digital risk score |

Classification rule: multiple features within one functional domain remain one domain (e.g., gait speed + stride variability + turning = Gait and mobility). Studies integrating two or more genuinely distinct functional domains may be classified as Multidomain.

# **Supplementary Table 2. Evidence and Methodological Characteristics of the Included Studies**

| **Author / Year** | **Evidence Type** | **Target / Comparison** | **Main Findings** | **Validation** | **Key Limitations** |
| --- | --- | --- | --- | --- | --- |
| Guarín et al., 2025 (11) | Cross-sectional discrimination | iRBD vs HC | ↑ amplitude/speed decrement in iRBD; RF balanced accuracy 79.8% | LOSO-CV | Small sample; no external validation |
| Arora et al., 2018 (12) | Cross-sectional discrimination | iRBD vs HC | Sens 91.9%, Spec 90.0%; voice most discriminatory | 10-fold CV; LOSO/LOO sensitivity analyses | No external validation; LOSO instability; possible spectrum/measurement bias |
| Arora et al., 2021 (13) | Cross-sectional discrimination | iRBD vs HC | Sens 60.7%, Spec 69.6%; speech features also associated with symptom severity | LOSO-CV | Single cohort; no external validation; sustained vowel only |
| Bertram et al., 2026 (14) | Cross-sectional discrimination | iRBD vs HC | Internal AUROC 0.96; Local test 0.86; OPDC 0.84; PACE 0.97 | Nested CV + blinded holdout + external validation + LODO-CV | Heterogeneous cohorts; single-night PSG reference; potential medication/comorbidity confounding |
| Bouhadoun et al., 2026 (15) | Longitudinal progression monitoring / phenoconversion association | iRBD progression; converters vs non-converters | Bradykinesia worsened over 50 weeks (d=0.50); baseline score worse in converters (d=1.10) | Repeated-measures longitudinal modelling; sensitivity analyses | Small converter number; practice effects; single-center iRBD cohort |
| Brink-Kjaer et al., 2023 (16) | Cross-sectional discrimination | iRBD vs non-iRBD controls | Actigraphy AUC 0.977; Sens 95.2%, Spec 90.5% | Participant-level LOOCV | Small sample; no external validation; limited RBD-mimic representation |
| Rusz et al., 2022 (17) | Cross-sectional difference & longitudinal monitoring | iRBD-AOF vs iRBD-NOF vs HC | Lower F0SD in iRBD-AOF at baseline; F0SD worsened over 24 months only with abnormal DAT-SPECT | No predictive/model validation | Small subgroups; male-only sample; no external validation |
| Cavallo et al., 2019 (18) | Cross-sectional discrimination | IH vs HC vs PD | RF 3-class accuracy 0.79; IH showed intermediate motor performance | 10-fold CV | Small sample; no external validation; no longitudinal IH outcomes |
| Cen et al., 2024 (19) | Phenoconversion association | iRBD converters vs non-converters/HC | Greater baseline stride-length asymmetry associated with phenoconversion (adjusted HR 4.47, 95% CI 1.09–18.35) | Prospective clinical follow-up | Small sample; only 10 converters; no external validation; multiple testing uncorrected |
| Cen et al., 2026 (20) | Phenoconversion association & longitudinal monitoring | iRBD converters vs non-converters; PD vs DLB trajectories | Reduced gait pace/arm swing associated mainly with PD conversion; converters showed faster dual-task stride-length decline | Prospective follow-up; competing-risk & sensitivity analyses | Few converters; no external validation; multiple comparisons uncorrected |
| Chen et al., 2014 (21) | Cross-sectional difference | iRBD vs HC | iRBD showed greater sway variability and reduced sway smoothness under challenging conditions | No predictive/model validation | Small sample; controls not PSG-confirmed; no longitudinal phenoconversion data |
| Del Din et al., 2019 (22) | Prospective prediction of future PD diagnosis | Future PD converters vs non-converters | Greater baseline gait variability and asymmetry predicted shorter time to PD diagnosis; step velocity and step length deviated approximately 3–4 years before diagnosis | Prospective longitudinal follow-up; Cox and mixed-effects modeling; no external validation | Only 16 converters; different sensor models across visits; exploratory analyses without multiple-comparison correction; no external validation |
| Del Din et al., 2020 (23) | Cross-sectional discrimination | iRBD vs HC | iRBD showed reduced gait pace and rhythm and greater variability during longer walking bouts; gait measures provided moderate discrimination, improved when combined with olfaction | ROC/logistic regression; no external validation | Cross-sectional; modest sample; limited discrimination; no longitudinal phenoconversion confirmation |
| Elshehabi et al., 2026 (24) | Prospective prediction of future PD diagnosis / longitudinal monitoring | Future PD vs non-PD | Slower baseline peak angular velocity was associated with future PD diagnosis and diverged ~8.8 years before diagnosis; classification AUC=0.805 | 75/25 train-test split; 1,000 bootstrap resamples for tuning; naïve test-set evaluation | Only 23 PD events; low sensitivity/PPV; turning-angle algorithm limitation; no external cohort validation |
| Chan et al., 2026 (25) | Cross-sectional group differentiation / biological association | HC vs iRBD vs PD | Net PIPR-6s progressively attenuated across HC→iRBD→PD (23.8%→18.6%→13.3%, P<0.001); associated with disease status, circadian measures, and cognition | Sensitivity analyses excluding antidepressant/melatonin users | Cross-sectional; no phenoconversion prediction; no formal ophthalmological examination; medication/confounding effects |
| Feng et al., 2020 (26) | Phenoconversion-associated longitudinal evidence | Control vs iRBD vs α-synucleinopathy; iRBD converters vs non-converters | Napping, activity fragmentation and inactivity worsened across disease stages; converters showed more baseline napping, higher kAR and lower activity/M10 than non-converters (d=0.56–0.80) | Matched analyses; FDR correction; functional linear modeling; sensitivity analyses | No independent external validation; actigraphy reflects gross activity and lifestyle; heterogeneous conversion outcomes; medication effects |
| Hlavnička et al., 2017 (27) | Cross-sectional discrimination | iRBD motor-positive vs motor-negative; PD vs HC | Automated speech features detected subtle RBD abnormalities; RBD motor-status classification accuracy 70.0%, sensitivity 73.9%, specificity 66.7% | Leave-one-subject-out CV for PD–HC feature pattern | Single center; modest sample; RBD reference based on UPDRS motor status rather than phenoconversion; no external validation |
| Gunter et al., 2025 (28) | Biological classification / dopaminergic-status discrimination | Normal vs abnormal DaT-SPECT across iRBD/PD/HC | Smartphone-only XGBoost achieved sex-adjusted AUC 0.80; combined smartphone + MDS-UPDRS-III AUC 0.82; gait, tremor, voice and dexterity were most informative | Participant-stratified 5-fold CV; repeated CV; nested 3-fold tuning | Small sample; very high feature-to-sample ratio; no independent external validation; mixed cohorts/app versions |
| Gupta & Patel, 2025 (29) | Longitudinal progression monitoring | Prodromal-PD progression; Prodromal-PD vs HC | Pairwise composite detected significant progression in Prodromal-PD (−0.36 SD/year) while MDS-UPDRS III did not (0.03 SD/year; p<0.0005); Prodromal-PD also showed smaller/slower submovements | Participant-level 5-fold CV; test–retest reliability; longitudinal comparison | Short 1-year longitudinal window; small longitudinal control group; no independent external validation; heterogeneous prodromal definition |
| Jeancolas et al., 2022 (30) | Cross-sectional discrimination | iRBD vs HC; iRBD with mild motor symptoms vs HC | iRBD showed altered pause duration and speech rhythm; classification BA 63%, increasing to 70% in iRBD with mild motor symptoms | Supervised classifier fusion; classification variability reported | Modest iRBD sample; iRBD males only; groups not fully age-matched; no longitudinal phenoconversion or external validation |
| Illner et al., 2024 (31) | Cross-sectional discrimination | iRBD vs HC; secondary comparisons with early PD | Passive calls discriminated iRBD from HC with AUC 0.79; passive calls + active reading increased AUC to 0.85; imprecise vowel articulation and monopitch were sensitive iRBD features | LOOCV; comparison across passive smartphone calls, active smartphone reading and laboratory recordings | Small iRBD sample; single-center; feature combination selected by exhaustive search; no independent external validation; participation/technology and privacy-related selection |
| Skrabal et al., 2022 (32) | Cross-sectional between-group difference | iRBD vs HC; iRBD olfactory/DAT-SPECT subgroups | VSA was significantly smaller in iRBD than HC (p=0.01); greater impairment occurred in iRBD with abnormal olfaction and abnormal DAT-SPECT | Between-group statistical testing; association with olfactory function and DAT-SPECT; no predictive/external validation | Cross-sectional; male-only sample; no classification performance or phenoconversion follow-up |
| Ma et al., 2021  (33) | Cross-sectional between-group difference | PSG-confirmed iRBD vs HC | iRBD showed reduced sagittal trunk range of motion (p=0.004), reduced peak angular velocity (p=0.001), and longer step time before turning (p=0.035); conventional spatiotemporal gait measures did not differ | Repeated-measures analysis across 3 walking conditions; covariate-adjusted analyses; no external/predictive validation | Small sample; cross-sectional; sex imbalance; no phenoconversion follow-up |
| Maremmani et al., 2018 (34) | Longitudinal progression monitoring | Idiopathic hyposmia monitored for subclinical motor deterioration | 1/20 hyposmic participants showed >15% deterioration in 6/8 motor parameters over 1 year; sensor abnormalities improved >15% with L-Dopa and worsened after withdrawal; subsequent DaTscan showed bilateral striatal DAT reduction | HC test–retest showed significant correlations (p<0.01); multimodal confirmation in one suspected case | Very small pilot sample; only 1 suspected preclinical PD case; arbitrary >15% thresholds; no established normative values; selective DaTscan verification; no confirmed PD phenoconversion |
| Massimi et al., 2026 (35) | Cross-sectional screening / discrimination | vPSG-confirmed iRBD vs controls | Actigraphy XGBoost: AUC 0.88 (95% CI 0.84–0.92), sensitivity 82.1%, specificity 83.5%; two-stage questionnaire + actigraphy screening achieved sensitivity 73.3% and specificity 100% | Fully nested 10-fold outer/5-fold inner stratified group CV with participant-level separation; out-of-sample evaluation | Retrospective case–control design; no independent external validation; heterogeneous cohorts/protocols; only subset had both modalities; possible awareness/selection bias; uncertain population-screening generalizability |
| Mirelman et al., 2016 (36) | Cross-sectional between-group difference | Nonmanifesting LRRK2-G2019S carriers vs noncarriers | Under dual-task walking, carriers showed greater arm-swing asymmetry (p=0.020), variability (p=0.038), reduced smoothness (p=0.006), and greater axial jerk (p=0.04); stride-time variability was also increased | Multicenter standardized protocol; adjusted mixed models with multiple-comparison correction; no longitudinal/external predictive validation | Cross-sectional; enriched convenience sample; incomplete LRRK2 penetrance; cannot distinguish prodromal changes from genotype-associated traits; medication data incomplete |
| Mirelman et al., 2011 (37) | Cross-sectional between-group difference | Asymptomatic LRRK2-G2019S carriers vs noncarriers | Carriers showed greater stride-time variability during dual-task walking (1.82% vs 1.26%, p=0.018) and altered variability during fast walking; dominant-frequency peak amplitude was lower in carriers during fast walking (p=0.005); usual gait measures were largely similar | Blinded gait assessment; repeated-measures analysis; Bonferroni correction; no longitudinal/external predictive validation | Small sample; cross-sectional; incomplete penetrance; single genetic population; observed abnormalities may represent an endophenotype rather than prodromal PD |
| Ophey et al., 2025 (38) | Clinical association / clinical-score estimation | Associations of accelerometry-derived PA, sleep and circadian features with motor/non-motor clinical outcomes in iRBD; secondary iRBD vs HC comparison | PA, sleep and circadian measures showed limited group-level differences; within iRBD, greater PA and more stable circadian rhythms were generally associated with lower non-motor symptom burden. Adding digital features significantly improved estimation of global cognition and memory scores | Elastic-net models with nested 10-fold CV; age-adjusted correlations with FDR correction | Cross-sectional design; modest single-center sample; no external validation; potential reverse causality; HC lacked PSG confirmation; GGIR sleep estimates may be imperfect for iRBD |
| Rovini et al., 2018 (39) | Cross-sectional discrimination | Three-class classification: IH vs PD vs HC; secondary two-class analyses: PD vs HC and PD vs HC+IH | RF performed best overall; three-class IH/PD/HC classification using both feet achieved accuracy 0.77, with IH recall 0.633; PD vs HC classification reached accuracy 0.967; discrimination between IH and HC remained limited | 10-fold CV; no independent external validation | Small single-center sample; cross-sectional; no phenoconversion follow-up; IH is a heterogeneous risk state and only a minority may develop PD; no independent external validation |
| Rusz et al., 2018 (40) | Cross-sectional discrimination | RBD vs HC; PD vs RBD/HC; secondary RBD-pPD vs RBD-A | Smartphone-derived F0 SD, DPI and RST distinguished RBD from HC; combined features yielded AUC 0.69 for RBD vs HC, 0.78 for PD vs RBD, and 0.85 for PD vs HC; no significant differences between RBD-pPD and RBD-A | LOOCV for logistic-regression classification; smartphone features cross-device compared with professional microphone; no independent external validation | Cross-sectional; modest sample; no phenoconversion follow-up; limited RBD-vs-HC discrimination; single smartphone model and controlled quiet environment; substantial cohort overlap with previous speech study |
| Pelicioni et al., 2026 (41) | Prospective association with future PD diagnosis | Incident future PD vs individuals without PD; incident PD additionally stratified by time to diagnosis | Real-world gait differences were detectable years before PD diagnosis; five measures were consistently associated with subsequent PD diagnosis: fewer daily steps, slower maximum walking speed, altered/lower step regularity, proportion of long walking bouts, and greater time walking with static arm positions; differences were detectable up to 6.8 years before diagnosis | Prospective follow-up; Cox regression | Single accelerometry assessment; PD diagnoses based on linked health records; no independent predictive-model validation. |
| Schalkamp et al., 2023 (42) | Future PD diagnosis prediction | Prodromal PD vs general population; diagnosed PD vs controls | Accelerometry-based models showed superior performance compared with genetics, lifestyle, blood biomarkers and prodromal symptoms. Reduced acceleration was detectable years before diagnosis. Models identified prodromal PD up to 7 years before clinical diagnosis. | Five-fold outer cross-validation; TRIPOD-guided prediction modelling; time-dependent AUROC analysis | No external validation; UK Biobank selection bias; PD diagnosis based on available clinical records without independent validation; accelerometry measured only once for 7 days; not suitable as diagnostic replacement |
| Šimek et al., 2026 (43) | Longitudinal progression monitoring | Longitudinal speech change in iRBD and early PD vs HC over 24 months | In iRBD, spectral mean index (β=-2.93, p<0.01) and neural embedding index (β=-3.96, p<0.001) showed greater decline than HC; vowel articulation (FRI, β=-2.62, p<0.01) and articulation rate (NSR, β=-3.16, p<0.001) also declined significantly | Longitudinal repeated-measures / linear mixed-effects modeling | Small single-country cohort; no phenoconversion prediction; smartphone/system-specific acquisition; passive call quantity varied between participants; no independent external validation |
| Simonett et al., 2021 (44) | Cross-sectional discrimination | PD vs HC; idiopathic anosmia vs HC; PD vs anosmia | SMART parameters detected bradykinesia-related abnormalities in PD; velocity and frequency-based models showed best discrimination; anosmia group showed intermediate impairment patterns suggesting possible prodromal motor changes | ROC analysis; correlation with MDS-UPDRS III finger tapping scores; no external validation | Small sample; cross-sectional; no longitudinal conversion follow-up; smartphone/video acquisition variability; anosmia group heterogeneous and not all will develop PD |
| Stefani et al., 2018 (45) | Cross-sectional screening / discrimination | iRBD vs controls; iRBD vs other sleep-related motor disorders | Quantitative actigraphy parameters distinguished iRBD from controls but not from other sleep-related motor disorders. Expert visual analysis of actigraphy patterns identified vPSG-confirmed iRBD with sensitivity 85–95%, specificity 79–91%, accuracy 81–91%, AUC 0.874–0.933 | Blinded expert ratings against vPSG-confirmed diagnosis | Small sample; cross-sectional design; expert-dependent interpretation; no automated algorithm; no longitudinal conversion to PD |
| Tzfoni et al., 2026 (46) | Cross-sectional screening / discrimination | Detect PSG-confirmed iRBD from home wearable data | Lumbar wearable sensor + ML identified iRBD with high sensitivity; SVM achieved sensitivity 93.33%, specificity 72.41%, accuracy 76.71%; performance plateau after five nights | vPSG reference standard; subject-level LOOCV; nested preprocessing and feature selection | Small sample; no external validation; moderate specificity; not prediction of PD conversion |
| Winer et al., 2023 (47) | Cross-sectional discrimination | iRBD vs healthy controls | iRBD showed lower circadian amplitude, reduced mesor and daytime activity (M10), and increased nighttime activity (L5), indicating altered 24-h rest–activity patterns | Clinical diagnosis of iRBD based on ICSD-3; matched healthy controls; sensitivity analysis using first 7 days of recordings | Cross-sectional design; no PD phenoconversion prediction; control data derived from UK Biobank; different geographic/time periods; no external validation |
| Zatti et al., 2024 (48) | Cross-sectional discrimination | iRBD vs HC; PD vs HC; iRBD vs PD | Digital turning assessment detected subtle mobility alterations in iRBD despite absence of subthreshold parkinsonism; iRBD showed longer turning duration and lower mean/peak angular velocity than controls during normal-speed TUG | PSG-confirmed iRBD diagnosis; drug-naïve PD confirmed by clinical assessment and dopaminergic imaging; age/sex-adjusted statistical analysis | Cross-sectional; no longitudinal conversion prediction; small iRBD sample; supervised clinical assessment rather than unsupervised home monitoring; external validation absent |
| Winer et al., 2024 (49) | Prospective association with future PD diagnosis | Future incident PD diagnosis | Lower activity rhythm amplitude, mesor and M10 were associated with increased PD risk; lower L5 also specifically predicted PD risk; fPCA identified reduced daytime activity component in future PD converters | Large population cohort; longitudinal follow-up; Cox regression adjusted for age, sex, education, health, BMI and socioeconomic status | Not a clinically defined prodromal PD cohort; PD diagnosis based on ICD/self-report records; lack of disease biomarkers; no direct conversion prediction among high-risk groups |
| Xie et al., 2026  (50) | Longitudinal prediction | Future PD diagnosis vs participants without PD diagnosis; prodromal PD group defined as individuals diagnosed >2 years after accelerometry measurement | Wrist-worn accelerometry combined with pretrained activity/sleep models and behavioral transition features improved prediction of time to PD diagnosis. The proposed XGBoost-Cox model achieved C-index 0.817 and mean AUC 0.855, outperforming conventional acceleration-based approaches. Sleep-stage transitions (e.g., N2→Wake) were among the most important predictive features. | UK Biobank longitudinal follow-up; 5-fold stratified cross-validation; performance evaluated using C-index, time-dependent AUC and PRAUC | PD diagnoses derived from linked health records without clinical validation; population-based cohort rather than clinically confirmed prodromal PD cohort; possible cohort overlap with previous UK Biobank accelerometry studies; external validation required |
| Prasuhn et al., 2021 (51) | Cross-sectional discrimination | Unaffected heterozygous Parkin mutation carriers vs mutation-free controls | Challenging postural and gait tasks discriminated unaffected Parkin mutation carriers from controls; best balanced accuracy reached 86% for posturography and 87% for gait analysis. | Internal classification analysis; no independent external validation | Small sample relative to feature dimensionality; limited model interpretability; cross-sectional design; no longitudinal phenoconversion assessment. |
| Sanchez-Rodríguez et al., 2022 (52) | Cross-sectional discrimination | Asymptomatic LRRK2 G2019S carriers vs healthy controls; PD groups for comparison | Sensor-based gait analysis detected subtle abnormalities in asymptomatic carriers mainly during fast walking. Increased step time, reduced step-time variability and altered asymmetry were observed. Neural network classified asymptomatic carriers vs controls with 82.5% accuracy. | Leave-one-out cross-validation (LOOCV) | Small sample; no correction for multiple comparisons; LOOCV may limit generalizability; longitudinal validation lacking. |
| Schalkamp et al., 2025 (53) | Risk stratification / biomarker association | PD vs HC; at-risk individuals evaluated against MDS prodromal criteria, DaTscan and α-synuclein SAA | Smartwatch-derived digital risk score distinguished PD from controls (AUPRC 0.96±0.01) and was associated with prodromal markers, reduced dopaminergic function and α-synuclein pathology-related markers | Nested 5-fold stratified cross-validation; evaluation in unseen at-risk cohort | No actual phenoconversion outcome; precision lower than sensitivity; possible bias toward individuals with mild motor impairment; external validation needed |

**Supplementary Table 3. Detailed Methodological Characteristics of Machine-Learning and AI-Based Studies**

| **Author / Year** | **Sample Size / Events** | **Feature Dimensionality** | **Feature Selection** | **Validation Strategy** | **Class Imbalance / Leakage Safeguards** | **Cohort Overlap** | **Key Methodological Limitations** |
| --- | --- | --- | --- | --- | --- | --- | --- |
| Guarín et al., 2025 (11) | 66; iRBD=16 | 4 features | Backward selection | LOSO-CV; no external validation | SMOTE and feature selection within training folds | Larger NIH-funded cohort; Potential cohort overlap; not confirmed | Small sample; potential overfitting; no external validation |
| Arora et al., 2018 (12) | 522; iRBD=104 | 998 → top 30 | 5 methods + majority voting | 10-fold; LOSO; LOO; no external validation | Equal-number resampling between groups | OPDC Discovery cohort | High dimensionality; LOSO instability; no external validation; uncontrolled home measurements |
| Arora et al., 2021 (13) | 539; iRBD=112 | 337 features | RF importance ranking | LOSO-CV; no external validation | Class-specific RF vote cut-offs; participant-level LOSO | Oxford Discovery Cohort; overlaps Arora et al., 2018 | High dimensionality; single cohort; uncontrolled recording quality; sustained vowel only |
| Bertram et al., 2026 (14) | Multicenter; training n=78 + independent test/external cohorts | Multiple handcrafted features; ~30 retained in final models | Ensemble ranking: MRMR + Boruta + correlation-based methods | 5×5 nested CV; blinded local test; 2 external cohorts; LODO-CV | Stratified group splitting; SMOTE; participant-level separation | OPDC overlap with earlier Oxford iRBD studies | Single-night PSG labels; cohort heterogeneity; medication/comorbidity effects; limited subgroup analyses |
| Brink-Kjaer et al., 2023 (16) | 84; iRBD=42 | Multiple handcrafted features | Data-driven feature/model comparison | Participant-level LOOCV; no external validation | All nights of held-out participant excluded from training | Stanford iRBD cohort; Potential cohort overlap; not confirmed | Small sample; no external validation; limited RBD mimics |
| Cavallo et al., 2019 (18) | 90; IH=30 | 48 features/limb → 27–31 | Univariate tests + correlation filtering | 10-fold CV; no external validation | Balanced groups; feature-selection nesting unclear | IPMP-MS/SensHand cohort; overlap likely | Small sample; possible selection leakage; no longitudinal confirmation of PD conversion |
| Elshehabi et al., 2026 (24) | 924 survival sample; 23 PD events | Low; 7 predefined turning measures | Cox screening; significant predictor entered into XGBoost with age/sex | 75/25 train-test split; 1,000 bootstrap tuning within training set; naïve test-set evaluation; no external validation | Severe class imbalance; PPD up-sampled in training set; independent test set retained | TREND cohort; overlaps Del Din et al., 2019 | Very few PD events; low PPV/sensitivity; no external validation; possible selection/model optimism; turn-angle measurement limitation |
| Hlavnička et al., 2017 (27) | 130 total; 50 iRBD | Moderate; 12 predefined acoustic features | Sequential search for best PD–HC feature combination | Leave-one-subject-out CV for PD–HC model; RBD subsequently classified; no external validation | Groups moderately balanced; subject-level CV used | Prague iRBD speech cohort; Potential overlap with later Prague/Rusz speech studies; not confirmed | Feature-combination optimization may inflate performance; motor-status surrogate outcome; single language/center; no phenoconversion follow-up |
| Gunter et al., 2025 (28) | 93 participants; 100 DaT scans; 52 normal / 48 abnormal | Very high; 1,058 smartphone features | SHAP-based feature ranking; top 10–500 features evaluated | Participant-stratified 5-fold CV; 5× repeated CV; nested 3-fold hyperparameter tuning | DaT classes balanced; participant-level split prevented repeated-recording leakage | OPDC Discovery cohort + Genoa; overlaps previous OPDC smartphone studies | High feature/sample ratio; feature-selection nesting unclear; no independent external validation; small HC sample; app/device heterogeneity |
| Jeancolas et al., 2022 (30) | 256 total; 41 iRBD / 117 PD / 98 HC | Moderate–high; high-level acoustic + low-level representations | Significant high-level features selected; multiple classifier representations combined | Supervised classification with classifier fusion; no independent external validation reported | Moderate group imbalance; participant-level leakage safeguards not clearly reported in main text | ICEBERG cohort; overlap with other ICEBERG speech studies possible | Small male-only iRBD group; age imbalance; feature/model-selection optimism possible; no phenoconversion follow-up; no external validation |
| Massimi et al., 2026 (35) | 396 overall; actigraphy 236 (78 iRBD/158 controls); two-stage n=129 | High: 113 candidate actigraphy features | Predefined/interpretable movement-feature framework; XGBoost feature importance | Fully nested 10-fold outer + 5-fold inner stratified group CV; no independent external validation | Participant-level group splitting; all nights from one participant kept within one fold; preprocessing/tuning/threshold selection restricted to training data; moderate class imbalance | Potential overlap with prior Stanford/Mount Sinai iRBD actigraphy cohorts | Retrospective case–control design; inter-cohort heterogeneity; partial data overlap; selection/awareness bias; no population-based external validation |
| Rovini et al., 2018 (39) | 90 total: 30 IH / 30 PD / 30 HC; no phenoconversion events | Moderate; 23 features per limb initially; reduced to 10–12 features per limb depending on dataset | Univariate significance testing followed by Spearman correlation filtering (ρ > 0.85) | 10-fold CV; no independent external validation | Balanced three-class dataset; participant-level CV structure not explicitly reported; feature selection appears to have been performed before CV, creating potential information leakage | IPMP-MS idiopathic-hyposmia cohort; potential overlap with Maremmani et al., 2018 and related SensHand/SensFoot studies | Small cross-sectional sample; potential feature-selection leakage; no external validation; no phenoconversion follow-up; IH heterogeneity and uncertain progression to PD |
| Schalkamp et al., 2023 (42) | Accelerometry cohort: 103,712; prediction subset: prodromal PD n=113; diagnosed PD n=153; general population n=33,009 | 82 accelerometry features; combined model 168 predictors including genetics, blood biomarkers, lifestyle and prodromal symptoms | LASSO regularization for feature selection; important predictors identified within models | Five-fold outer cross-validation; TRIPOD reporting framework; survival random forest validation | Severe class imbalance; AUPRC selected because outcome prevalence was low; class weighting applied to prioritize sensitivity | UK Biobank accelerometry cohort (overlaps with Williamson 2021, Pelicioni 2026 and other UKBB wearable studies) | Single 7-day measurement; no external replication; diagnosis derived from UKBB records without clinical validation; prediction performance may not generalize outside UKBB |
| Tzfoni et al., 2026 (46) | n=73; iRBD=15; control=58; no PD conversion events | 126 features/night; reduced to 112 after correlation filtering | Recursive feature elimination (RFE); SHAP identified important features | Subject-level LOOCV; nested preprocessing; normalization, feature selection and model training within each fold | Nested pipeline avoided data leakage; groups imbalanced (15 vs 58) but subject-level validation used | Recruited from Genetics in Parkinson’s Project cohort; possible overlap with related LEMON cohort studies | Small exploratory proof-of-concept study; LOOCV variance; lack of external validation; false positives; wearable cannot determine sleep architecture |
| Xie et al., 2026 (50) | Large UKBB accelerometry cohort; 383 future PD diagnoses >2 years after accelerometry | High; 360 input features | Hierarchical Markov transition feature extraction; ablation analysis; no conventional feature selection reported | Event-stratified 5-fold CV; C-index, time-dependent AUC and PRAUC; no independent external validation | Severe event imbalance; event-stratified CV and PRAUC used; no specific resampling reported | UK Biobank accelerometry cohort; overlaps other UKBB wearable studies | Rare PD events; high feature dimensionality; no independent external validation; record-based PD diagnoses not clinically validated; population-based rather than formally defined pPD cohort |
| Prasuhn et al., 2021 (51) | n=51; Parkin mutation carriers=27; controls=24; no phenoconversion events | Multiple sensor-derived gait and posturography motion metrics; exact feature number not specified | No predefined reduction; GBT models used to handle high-dimensional sensor features | Double-blinded assessment; GBT classification; balanced accuracy and ROC-AUC evaluation | Groups relatively balanced; no ML leakage procedure explicitly reported | CHRIS population-based genetic cohort; overlap with previous Parkin carrier studies possible but not confirmed | Small exploratory cohort; cross-sectional; genetic high-risk model rather than clinically defined prodromal PD; feature interpretability limited; no external validation |
| Sanchez-Rodríguez et al., 2022 (52) | n=27 asymptomatic LRRK2 carriers; n=36 controls; no phenoconversion events | Multiple spatiotemporal and kinematic gait variables derived from 16 IMU sensors; exact number not reported | Variables generated from gait parameters; no explicit feature reduction reported before neural network modelling | Leave-one-out cross-validation for neural network classification | Groups moderately balanced; no explicit leakage prevention procedure reported | Same research network as previous LRRK2 G2019S gait studies; possible cohort overlap cannot be excluded | Small exploratory cohort; cross-sectional; genetic carrier state does not guarantee conversion; limited generalizability; no external validation |
| Schalkamp et al., 2025 (53) | PD n=143; HC n=34; at-risk evaluation cohort n=109 | 10,962 time-series features extracted from 14 digital measures | Elastic-net regularization selected predictive features | Nested 5-fold stratified cross-validation with inner grid search | At-risk cohort excluded from model training and validation to reduce leakage | PPMI cohort; possible overlap with previous PPMI digital studies | Lack of longitudinal conversion validation; heterogeneous at-risk population; precision limitations; external validation required |

**Supplementary Table 4. Full-text articles excluded after eligibility assessment and primary reasons for exclusion**

| **Author / Year** | **Article Title** | **Primary Reason for Exclusion** |
| --- | --- | --- |
| Abdelfattah et al., 2025 | Automated Detection of Isolated REM Sleep Behavior Disorder Using Computer Vision | Concept/objective not eligible, focused on the detection of iRBD itself rather than PD-related characterization, risk stratification, or prediction. |
| Cooray et al., 2021 | Proof of Concept: Screening for REM Sleep Behaviour Disorder with a Minimal Set of Sensors | Concept/objective not eligible, focused primarily on screening and detection of RBD rather than PD-related digital biomarkers or outcomes. |
| Perkins et al., 2021 | Saccade, Pupil, and Blink Responses in Rapid Eye Movement Sleep Behavior Disorder | Device not eligible, digital measures were obtained using a laboratory-based video eye-tracking system rather than an eligible wearable or portable device. |
| Riek et al., 2024 | Multimodal Oculomotor Assessment Reveals Prodromal Markers of Parkinson's Disease in Non-manifesting LRRK2 G2019S Mutation Carriers | Device not eligible, oculomotor measures were acquired using a laboratory-based eye-tracking system rather than an eligible wearable or portable device. |
| La Morgia et al., 2022 | Chromatic Pupillometry in Isolated Rapid Eye Movement Sleep Behavior Disorder | Device not eligible, pupillary measures were obtained using a laboratory-based pupillometry system rather than an eligible wearable or portable device. |
| Perkins et al., 2026 | Saccade, Pupil, and Blink Abnormalities in Prodromal and Manifest Alpha-Synucleinopathies | Device not eligible, oculomotor measures were obtained using a laboratory-based video eye-tracking system rather than an eligible wearable or portable device. |
| Possti et al., 2024 | Semi Automatic Quantification of REM Sleep Without Atonia in Natural Sleep Environment | Concept/objective not eligible, focused primarily on RSWA quantification and RBD detection rather than PD-related characterization or prediction. |
| Šubert et al., 2022 | Linguistic Abnormalities in Isolated Rapid Eye Movement Sleep Behavior Disorder | Concept not eligible, biomarkers were based primarily on transcribed and linguistically annotated discourse rather than wearable- or portable-device-derived digital measures. |
